# Gut Microbiome Composition and Function, Diet, and Clinical Factors in Relation to Fermentable Carbohydrate-Induced Bloating: A Double-Blind, Randomized, Crossover Trial

**DOI:** 10.14309/ajg.0000000000003997

**Published:** 2026-03-17

**Authors:** Dominic N. Farsi, Aurélie Cotillard, Bridgette Wilson, Daniel So, Philippa S. Gibson, Rachael Slater, Chris Probert, Samantha Morris, S. Mark Scott, Laurent Quinquis, Matthieu Pichaud, Sudarshan Shetty, Julien Tap, Boris Le Nevé, Megan Rossi, Kevin Whelan

**Affiliations:** 1 Department of Nutritional Sciences, King's College London, London, United Kingdom;; 2 Danone Research & Innovation, Gif sur Yvette, France;; 3 University of Liverpool, Institute of Systems, Molecular and Integrative Biology, Liverpool, United Kingdom;; 4 Blizard Institute, Queen Mary University of London, London, United Kingdom;; 5 Danone Research & Innovation, Utrecht, the Netherlands.

**Keywords:** functional bloating, fermentable carbohydrates, fiber, gut microbiome

## Abstract

**INTRODUCTION:**

Specific foods are associated with abdominal bloating, which can significantly affect quality of life. To identify responders to fiber-induced bloating and the mechanisms underpinning clinical and microbial responses.

**METHODS:**

Double-blind, placebo-controlled, randomized, 2-period, 2-challenge crossover trial in 41 individuals with functional bloating. Participants were randomized to 8 g/d of fructan or α-galacto-oligosaccharides (α-GOS) for 7 days with a 21-day washout. Clinical, nutritional, microbial (shotgun sequencing, metatranscriptomics), and fermentation (short-chain fatty acids, volatile organic compounds, breath hydrogen) profiles were characterized before each challenge to identify factors predicting response and after the challenge to elucidate mechanisms underpinning food-induced bloating.

**RESULTS:**

Thirty-nine participants completed both challenges (39 fructan, 40 α-GOS). Overall, 7 (7/39, 17.9%) participants were fructan responders and 8 (8/40, 20%) were α-GOS responders (experienced fiber-related symptom induction). Clinical metrics indicative of bloating distinguished responders and nonresponders to both challenges, including greater abdominal girth (fructan, *P* = 0.009; α-GOS, *P* = 0.030). α-GOS responders had higher breath hydrogen (H_2_) prechallenge than α-GOS nonresponders (*P* = 0.011). Trends were identified within metagenomic and metatranscriptomic gut microbial analyses, with higher carbohydrate active enzyme (CAZyme) diversity in fructan responders (prechallenge, adjusted *P*-value (*P*adj) = 0.024; postchallenge, *P*adj = 0.042) and greater increase in gene expression for gamma-aminobutyric acid (GABA) degradation in α-GOS responders (*P*adj = 0.041).

**DISCUSSION:**

A higher burden of GI symptoms predicts clinical response to fermentable fibers in functional bloating, while for α-GOS, higher repeated fasting breath H_2_ is also a predictor. Gut microbiome function and fermentation is associated with functional bloating; however, further investigations are required to draw firm conclusions for the microbial influence in this interplay.

## Graphical abstract

**Figure:**
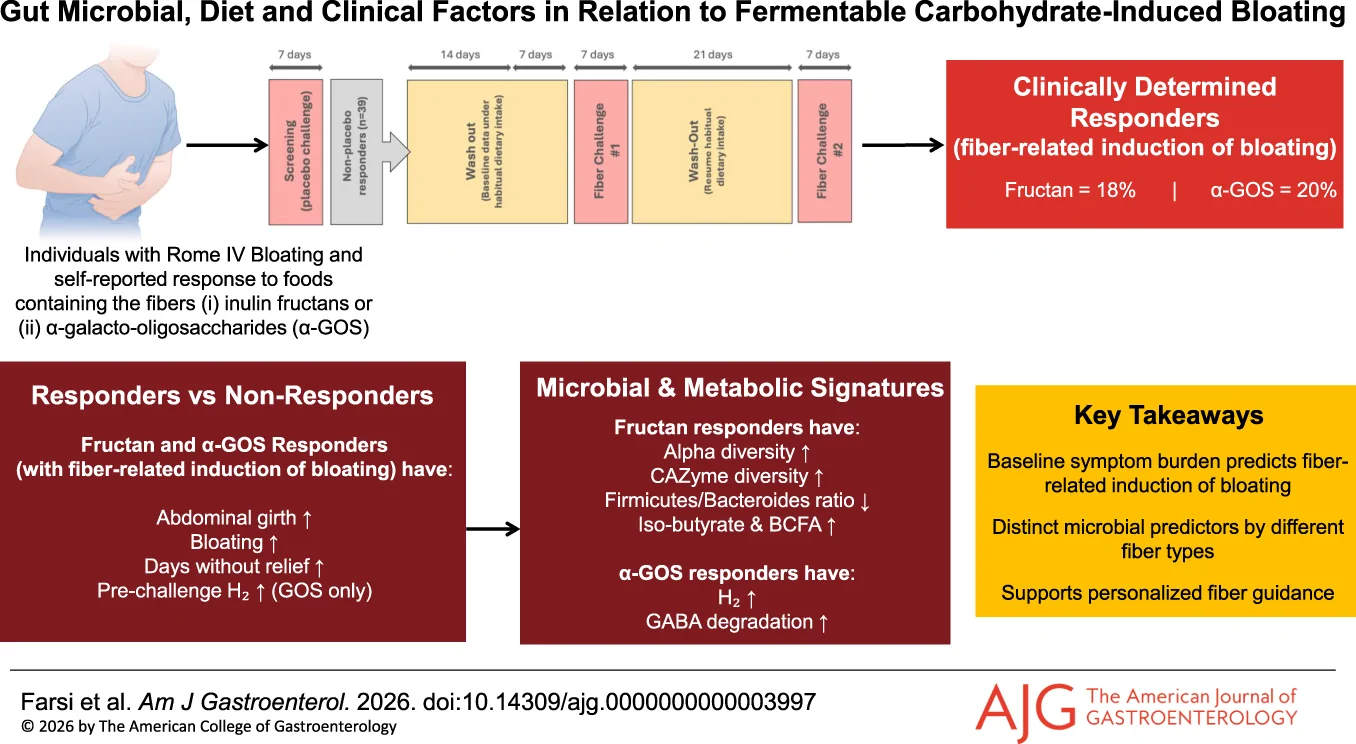

## INTRODUCTION

Variations in response to the same dietary component between individuals are well established (1–3). This has fueled interest in personalized nutrition and providing targeted nutritional advice based on individual characteristics, such as genotypic and phenotypic variation, including the gut microbiome. Diet and the gut microbiome have a bidirectional relationship, with foods having differing effects on the gut microbiome between individuals, while the microbiome may be a determinant of the impact of foods on health. Nevertheless, despite an increasing body of research, predicting how an individual's gut microbiome will respond to a given dietary intervention, and how this relates to the influence on health, at present, remains largely unknown.

Gastrointestinal (GI) symptoms are common amongst the general population (4), even in the absence of a diagnosed disorder of gut-brain interaction (DGBI). Abdominal bloating is a bothersome issue in the general population, with up to 30% of otherwise healthy individuals experiencing bloating which they attribute to diet (5–7). Functional bloating is a DGBI characterized by self-reported symptoms of recurrent abdominal fullness or pressure (in addition to mild pain and/or minor bowel movement abnormalities) with a prevalence of 3.5% (8). The mechanisms underlying functional bloating are yet to be fully elucidated. Some studies suggest the volume of gas contained in the gut can be a factor in symptom induction; however, data indicates that patients with bloating often manifest perturbed viscerosomatic reflex responses to gas distention (9,10), suggesting that visceral hypersensitivity (VH) rather than purely gas volume may play a role (11). Functional bloating can affect quality of life, displaying associations with higher levels of depression, anxiety, lower global mental and global physical health compared with individuals without bloating (4) and is reported to be related to diet quality (12). Diet is therefore linked with bloating in both the general population and in those specifically with functional bloating. Elucidating the mechanisms underpinning food-induced bloating and the ability to identify “responders” to specific foods will be an important advance.

Fermentable fibers are common triggers of food-induced symptoms, in particular the inulin-type fructans and α-galacto-oligosaccharides (GOS). Despite fulfilling criteria for prebiotics, both fructans and GOS can worsen GI symptoms in susceptible individuals, particularly inducing bloating (13). Inulin-type fructans are fructose polymers linked by β-2,1 bonds with a terminal α-linked glucose, with shorter polymers (degree of polymerization, DP, 3–8) termed oligofructose and longer polymers (DP < 60) termed inulin (14). GOS are polymers of galactose with a terminal glucose monomer. GOS consist of α-linked galactose, α-linked glucose, and a terminal β-linked fructose (e.g., raffinose, stachyose, and verbascose). Fructans are present in a wide variety of commonly consumed foods such as wheat, rye, garlic and onion, while GOS are found predominantly in legumes such as lentils, chickpeas and kidney beans (15,16), all of which can induce gut symptoms in some people with DGBI (17).

The aims of this study were threefold: (1) to identify people with functional bloating who experience symptom induction to fructans and GOS; (2) to characterize microbiome composition, function, and expression associated with experiencing bloating to these fibers, using both metagenomic and metatranscriptomic analyses; and (3) to elucidate the mechanisms underpinning both the clinical and microbial responses.

## METHODS

### Study design and participants

Pre-FIBRe (Predeterminants of Fibre-Induced Bloating Response) was a double-blind, placebo-controlled, randomized, 2-period, 2-challenge crossover trial in individuals with functional bloating. Further details regarding recruitment strategies and inclusion criteria are described in Supplementary Material (http://links.lww.com/AJG/D912).

Ethical approval was received from the Research Ethics Committee, King's College London (HR-19/20–18704), and all participants provided informed consent before participation. The trial was conducted in compliance with the Declaration of Helsinki (1996) and registered on ClinicalTrials.gov (NCT04802798).

### Procedures

The study comprised a glucose placebo challenge as part of the screening process (visit [V]1 and V2). Those who responded symptomatically to the inert challenge (nocebo responders) were excluded from further participation. Remaining participants then underwent 2 challenge periods in randomized order for 7 days with a 21-day washout between. Challenges were only performed once participants were experiencing adequate relief of bloating symptoms. The supplements were either 8 g/d fructan or 8 g/d GOS taken in 2 doses contained in sachets (4 g with first meal, 4 g with last meal of the day, each mixed with 300 mL of water). Sachets were packaged bespoke for this study in plain, identical sachets with no markings other than code and instructions for participants. Full details of the placebo challenge, randomization and blinding procedures as well as intervention products, dosages, and timings are included in the Supplementary Material (http://links.lww.com/AJG/D912).

During the challenge and washout phases of the trial, participants were instructed to maintain all dietary habits as normal, and to avoid their trigger foods. Adherence was measured by: (1) return of any unused product; (2) a standard question on adherence (in the past 7 days how many sachets have you taken?) at the end of each intervention; and (3) 7-day adherence diaries.

The study schema is presented in Figure 1.

**Figure 1.**
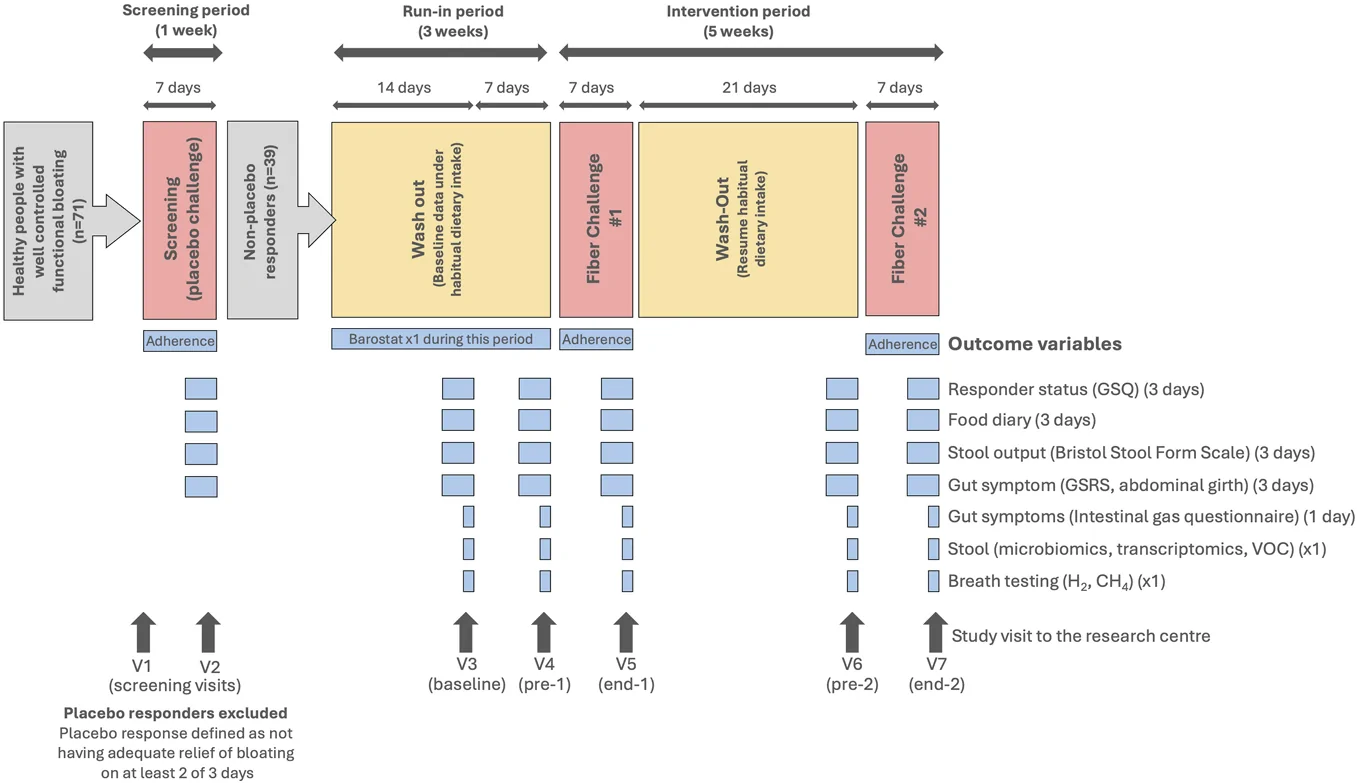
Study schema for the pre-FIBRe study.

### Outcomes

Outcomes were measured at baseline (V3), prechallenge 1 (V4), end of challenge 1 (V5), prechallenge 2 (V6) and end of challenge 2 (V7). The primary end point was bloating symptoms in response to the fiber challenges, which was measured for the final 3 days of the end of challenge periods (V5 and V7). “Responders” (those experiencing fiber-related induction of bloating) were defined as participants who experienced increased bloating during the fructan or GOS challenge. This was measured every day for the final 3 days of the fiber challenge using the Global Symptom Question (GSQ), “Today, did you have adequate relief of your bloating symptoms” (yes/no), which is a dichotomous response definition recommended by Rome due to its ease and validity (18). “Responders” (those experiencing fiber-related induction of bloating) were those reporting they did not have “adequate relief of bloating symptoms” (by responding “no” to the GSQ) on at least 2 of the final 3 days. All other participants were defined as “nonresponders” (those not experiencing induction of bloating) to a fructan or GOS challenge.

Outcomes included clinical, nutritional, microbial, and fermentation profiles which served as both determinants (prechallenge values) and mechanisms (end of challenge values; delta between pre and end of challenge values) underpinning the response to the fiber challenges (Figure 1).

### Clinical and nutritional analysis

Clinical profiles were determined using the following validated clinical questionnaires and tools: GSQ; Food diary; Gastrointestinal Symptom Rating Scale (GSRS) Questionnaire (19); abdominal girth (measured using a tape measure); Bristol Stool Form Scale (BSFS), daily using the final 3-days of a prospective diary (20); Intestinal Gas Questionnaire (IGQ, 1 day) (21); appetite (days 1 and 7 of the diary); and body fat (bioelectrical impedance analyzer, TANITA BC-418 MA). VH was measured during the baseline period using an electromechanical barostat; full details are provided in Supplementary Material (http://links.lww.com/AJG/D912).

Nutritional intake was measured during the final 3-days of the 7-day challenge period to coincide with symptom recording. Energy and nutrient values were calculated using Nutritics nutritional analysis software (Nutritics, Research Edition, v5.09, Dublin) based on standard UK food composition tables (McCance and Widdowson). The intake of fermentable oligosaccharides, disaccharides, monosaccharides, and polyols (FODMAP) was calculated using the Monash University FODMAP composition database (Monash University).

### Microbiome metagenomic sequencing and transcriptomics

On the day of a study visit, participants collected a whole fresh stool sample in a sealable sterile bag that was placed immediately on ice and delivered to the laboratory within 1 hour. Samples were homogenized in a stomacher for 2 minutes before aliquots were stored at −80 °C until analysis. Fecal microbiome composition and functional activity were assessed using shotgun metagenomic and metatranscriptomic sequencing, respectively. Full details of the gut microbial analyses are included in Supplementary Material (http://links.lww.com/AJG/D912).

### Fermentation (SCFA, VOC, breath gas)

Fecal short-chain fatty acids (SCFA) were quantified using gas liquid chromatography, and volatile organic compound (VOC) analysis was performed using gas-chromatography mass-spectrometry, both as previously described by Wilson et al (22). Fasting breath gas was measured at baseline (V3) and immediately before (V4, V6) and at the end of (V5, V7) each challenge period using a Gastrogenius Breath tracker (Laborie Medical Technologies; Enschede, The Netherlands). Hydrogen (H_2_) and methane (CH_4_) measures were interpreted using published criteria (23). Further details are included in Supplementary Material (http://links.lww.com/AJG/D912).

### Statistical analysis

#### Clinical, nutritional, and fermentation profiles analysis

Clinical, nutritional, and fermentation determinants of fiber response were assessed by unpaired t-tests or Mann-Whitney *U* tests depending on data distribution to compare the differences between responders and nonresponders prechallenge (V4/V6). For differences in end of challenge measures (mechanisms—part 1), analysis of covariance (ANCOVA) was performed with the corresponding prechallenge (V4/V6) metric included as a covariate. For differences in delta prechallenge to end of challenge (mechanisms—part 2), no covariates were considered to be appropriate, and unpaired t-tests or Mann-Whitney *U* tests were performed instead.

#### Microbiome analysis

Prechallenge (determinants, V4/V6) and end of challenge (mechanisms, V5/V7) comparisons between responders and nonresponders were assessed using unpaired t-tests or Mann-Whitney *U* tests based on distribution for alpha-diversity indices and the *Faecalibacterium*/*Bacteroides* ratio, PERMANOVA for beta-diversity indices and DESeq2 models with responder status effect for taxonomic and functional data. The same methods were used to assess baseline (V3) comparisons between VH and non-VH participants. To elucidate the difference in delta pre to end of challenge between responders and nonresponders (mechanisms, V5-V4 or V7-V6), variations between end of challenge and prechallenge of alpha-diversity indices and dissimilarities between end of challenge and prechallenge of beta-diversity indices were analyzed using unpaired t-tests or Mann-Whitney *U* tests. In addition, taxonomic and functional data were analyzed using DESeq2 models considering the subject effect and the interaction between time and responder status.

For all DESeq2 models, multiple testing adjustments were performed using the Benjamini-Hochberg procedure (24). Unless otherwise specified, *P*-values reported for microbiome and metatranscriptomic features correspond to Benjamini–Hochberg FDR–adjusted values derived from DESeq2 analyses and are denoted as “*P*adj” (in text, figures and supplementary tables).

#### Statistical details

All statistical analyses were performed in R 4.0.3 or R 4.2.1 (R Foundation for Statistical Computing, Vienna, Austria) and GraphPad Prism 9.4.1 (GraphPad Software Inc; Sand Diego, CA). *P* < 0.05 was considered statistically significant in two-sided tests. Full details of statistical analysis, including sample size and populations analyzed, are provided in Supplementary Material (http://links.lww.com/AJG/D912).

## RESULTS

### Participants

Fifty-seven participants completed the screening placebo challenge; 16 were excluded due to withdrawal (n = 7), symptom response during placebo (n = 6), prohibited medication use (n = 2), or unrelated food poisoning (n = 1). 41 participants were randomized, and 40 completed the first challenge. One participant withdrew before commencing due to protocol deviation, and another after the first challenge due to antibiotic use. In total, 39 participants completed both challenges. Thus, 39 were included in the modified intention to treat (mITT) population for the fructan challenge and 40 for GOS (Figure 2).

**Figure 2.**
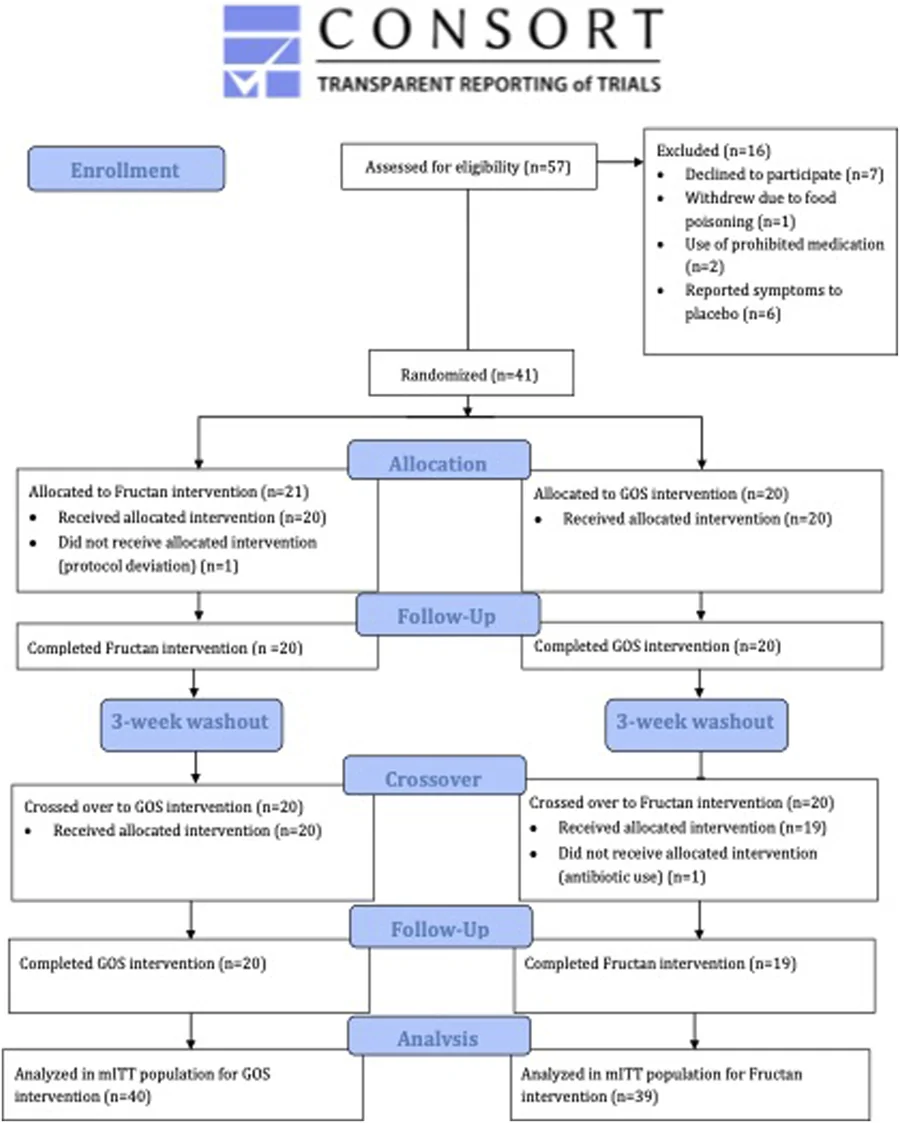
CONSORT diagram for the pre-FIBRe study.

### Response to fiber interventions

Based on the responder criteria applied (those experiencing fiber-related induction of bloating), 7/39 (17.9%) participants responded to fructans and 8/40 (20%) to GOS, with 3/39 (7.7%) responding to both. In line with our responder definitions, at the end of the challenge period, fructan responders reported more days with inadequate relief of bloating during fructan challenge (median 100% days, IQR 80–100, vs 0% days, 1–10, *P* < 0.001) and GOS responders reported more days with inadequate relief during GOS (median 70% days, IQR 70–100 vs 0% days 0–30, *P* < 0.001) than the respective nonresponders.

Baseline characteristics of responders and nonresponders to the fiber challenges are summarized in Table 1. Fructan responders had higher body fat mass than nonresponders (*P* = 0.035), with no other baseline differences.

**Table 1.**
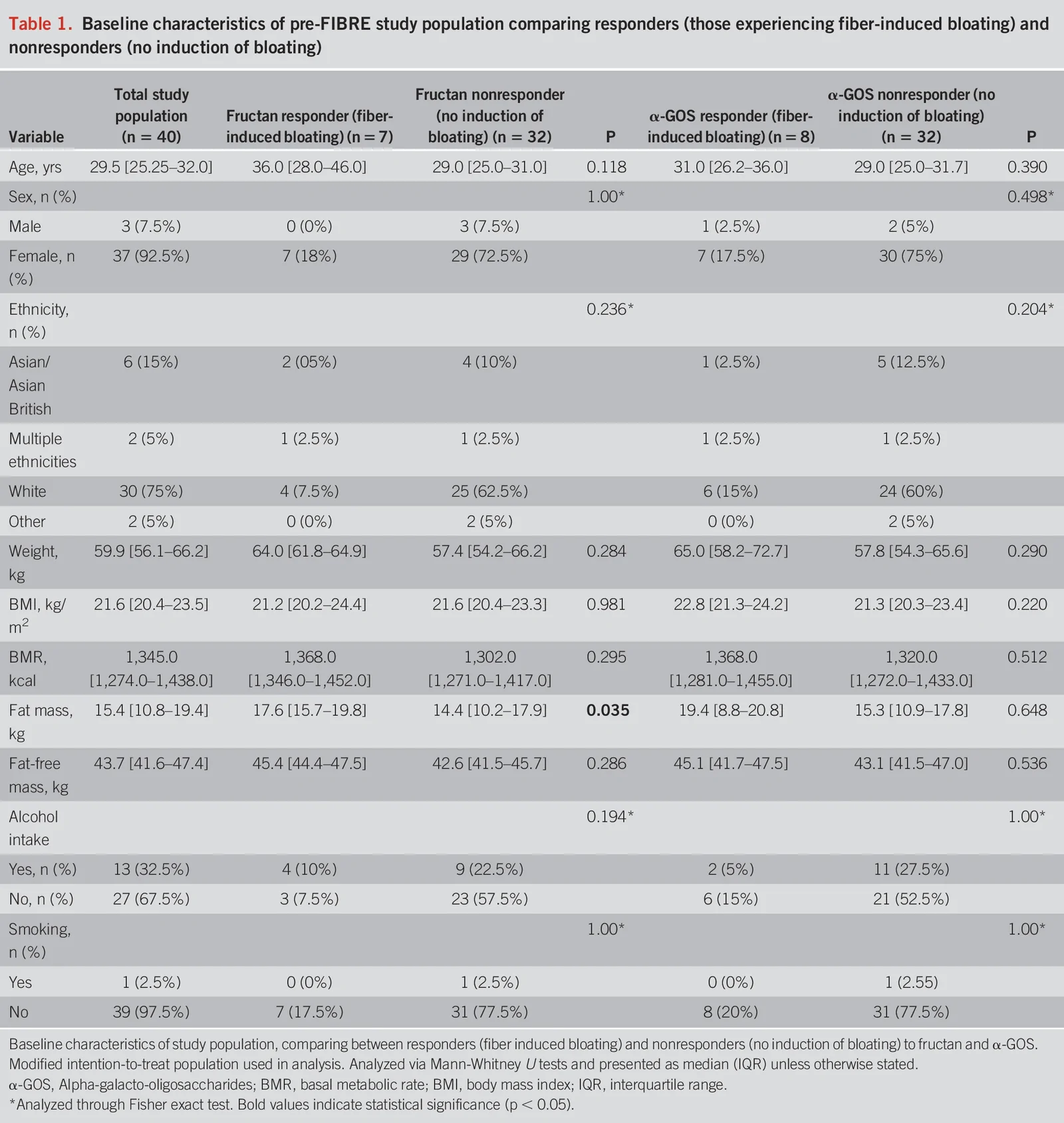
Baseline characteristics of pre-FIBRE study population comparing responders (those experiencing fiber-induced bloating) and nonresponders (no induction of bloating)

### Clinical symptoms and response to fibers

Fructan responders (those experiencing fiber-related induction of bloating) reported more days with inadequate relief prechallenge (*P* = 0.017), while postchallenge, they experienced greater symptom induction including higher scores for bloating (IGQ *P* = 0.003; GSRS *P* = 0.001), abdominal pain (GSRS *P* = 0.020), discomfort (*P* = 0.013), flatulence (*P* = 0.005), and overall symptoms (*P* = 0.018). They also showed greater abdominal girth (*P* = 0.009) and fewer normal consistency stools (*P* = 0.030).

For GOS, responders (those experiencing fiber-related induction of bloating) exhibited higher prechallenge bloating (GSRS, *P* = 0.011), and postchallenge, they experienced greater symptom induction including higher bloating (IGQ *P* = 0.018; GSRS *P* < 0.001), difficult gas evacuation (IGQ *P* = 0.002), incomplete stool evacuation (GSRS, *P* = 0.003), and overall symptoms (GSRS, *P* < 0.001). Abdominal girth was also higher (*P* = 0.030). Full comparisons are presented in Table 2, and delta values are presented in Supplementary Table 1 (http://links.lww.com/AJG/D912).

**Table 2.**
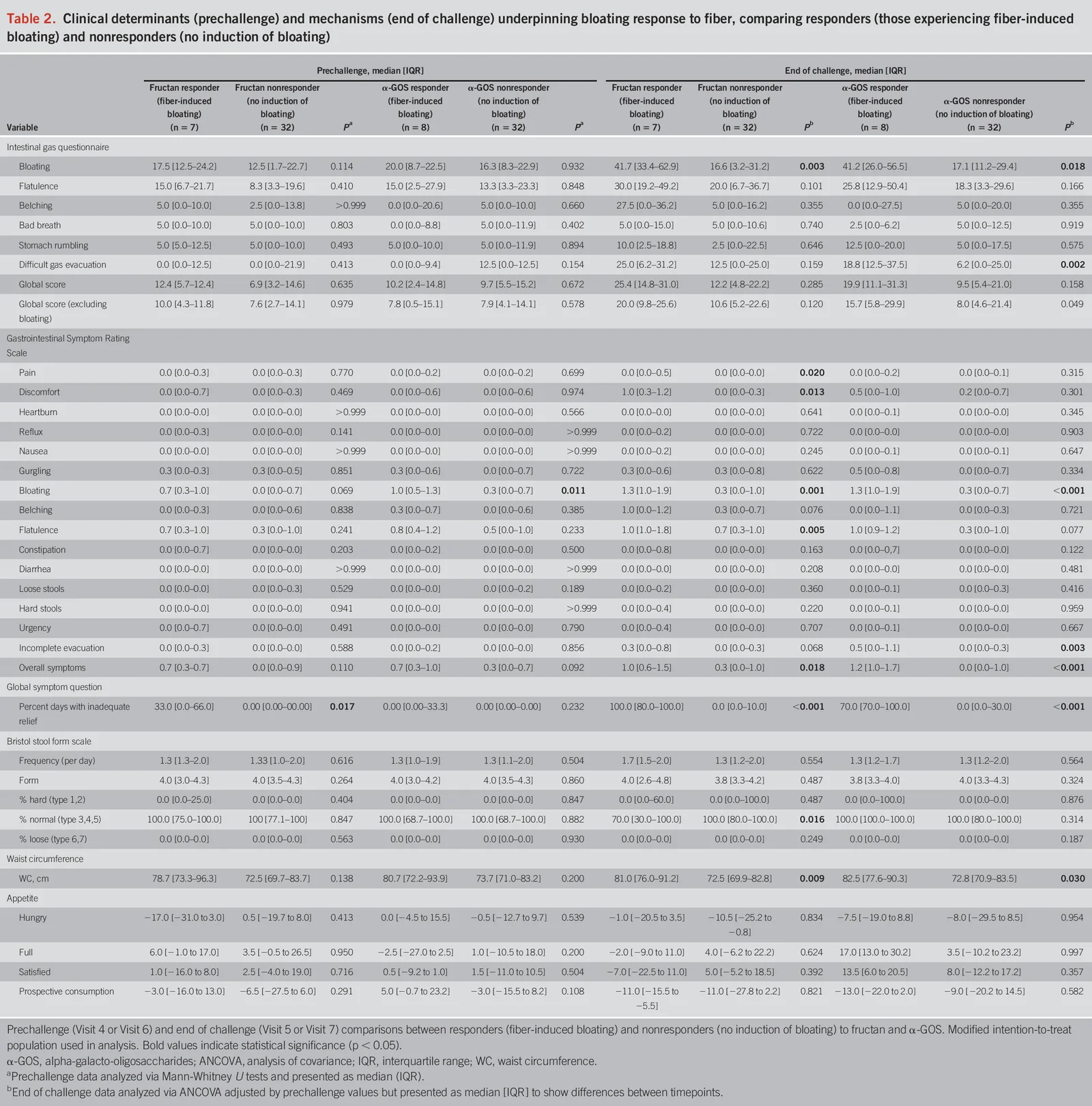
Clinical determinants (prechallenge) and mechanisms (end of challenge) underpinning bloating response to fiber, comparing responders (those experiencing fiber-induced bloating) and nonresponders (no induction of bloating)

### Nutrient intake and response to fibers

Fructan responders consumed more calcium (*P* = 0.039) and mannitol (*P* = 0.009) prechallenge. Postchallenge, they consumed more fiber (*P* = 0.017), nonstarch polysaccharides (NSP) (*P* = 0.001), potassium (*P* = 0.025), magnesium (*P* = 0.004), and mannitol (*P* < 0.001), and lower sodium (*P* = 0.008).

GOS responders consumed more protein (*P* = 0.019), potassium (*P* = 0.033), phosphorus (*P* = 0.018), FODMAPs (*P* = 0.049), lactose (*P* = 0.027), and energy (*P* = 0.035) prechallenge. Postchallenge, only fiber intake differed, being lower in responders (*P* = 0.018) (Table 3; Supplementary Table 2, http://links.lww.com/AJG/D912).

**Table 3.**
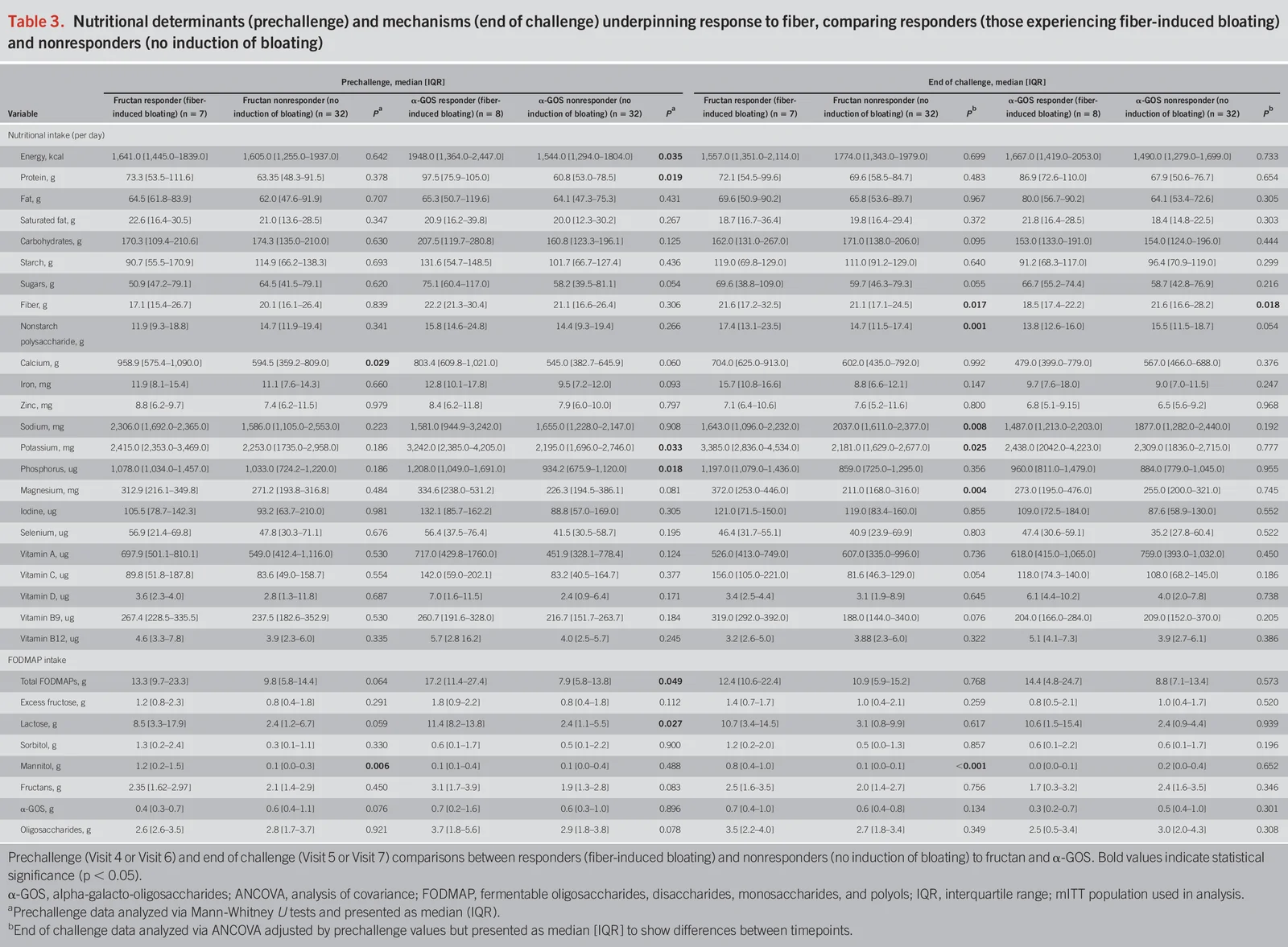
Nutritional determinants (prechallenge) and mechanisms (end of challenge) underpinning response to fiber, comparing responders (those experiencing fiber-induced bloating) and nonresponders (no induction of bloating)

### Microbiome, fermentation, and response to fibers

Higher prechallenge microbiome diversity (Shannon index for mOTUs and CAZymes, *P* = 0.035 and *P* = 0.024) characterized fructan responders (Figure 3). Shannon diversity and gene richness decreased postchallenge in responders (*P* = 0.025 and *P* = 0.035), although CAZyme Shannon index remained higher (*P* = 0.042). Few specific taxa were associated with response (Supplementary Figure 1, http://links.lww.com/AJG/D912); *Ruminococcus_C sp000980705* was less abundant in fructan responders both prechallenge and postchallenge (*P*adj < 0.001) and *Faecalibacterium sp900758465* only postchallenge (*P*adj = 0.0496) (Figure 4); a lower *Faecalibacterium*/*Bacteroides* ratio predicted response to both fructan (*P* = 0.008) and GOS (*P* = 0.015) (Supplementary Figure 7, http://links.lww.com/AJG/D912).

**Figure 3.**
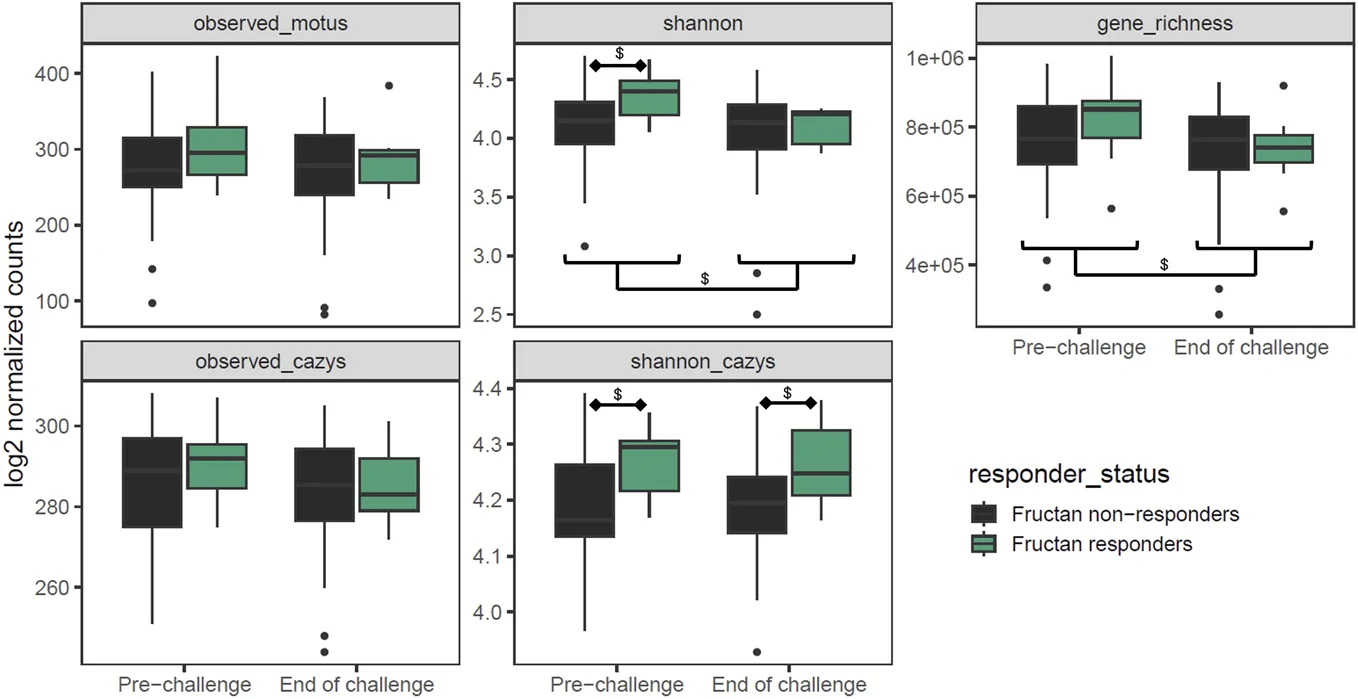
Boxplots of alpha diversity indices by fructan responder status. $*P* < 0.05 with T-tests of Mann-Whitney *U* tests depending on data normality for prechallenge, end of challenge, or delta challenge values.

Metatranscriptomic analysis showed lower expression of a few gut metabolic modules (GMMs) and several CAZymes (n = 32), including one cohesin CAZyme, mainly postchallenge (Supplementary Figures 2–3, http://links.lww.com/AJG/D912). Targeted check showed a trend for lower fructan degradation pathways and GH32 fructanase expression in responders (Figure 4).

**Figure 4.**
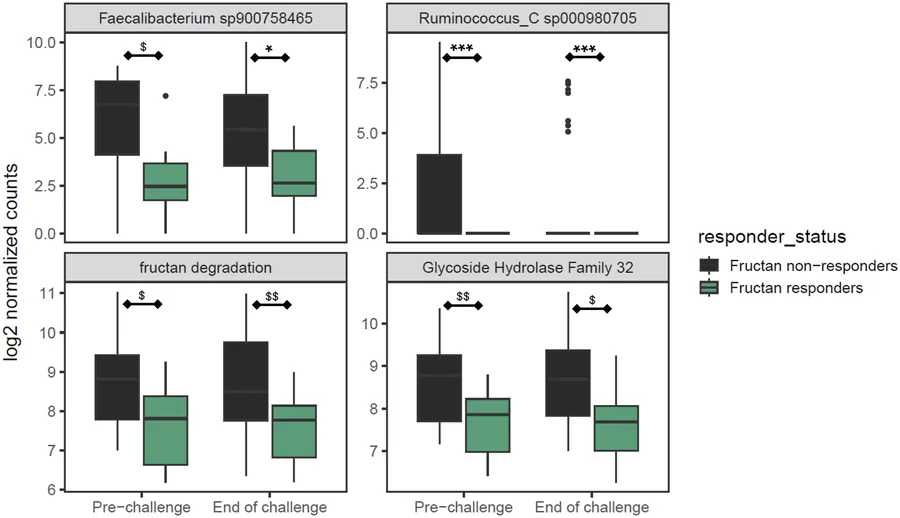
Boxplots of significant mOTUs (metagenomic) and of functions (metatranscriptomic) related to fructan degradation by fructan responder status. ***Padj < 0.001, *Padj < 0.05, $$*P* < 0.01, $*P* < 0.05 with DESeq2 analysis.

No diversity differences were seen for GOS. However, several mOTUs were associated with GOS response: *Sutterella wadsworthensis A, CAG-177 sp003514385*, and *CAG-83 sp003539495* were less abundant in responders prechallenge (*P*adj < 0.001), with the first 2 remaining significant postchallenge (*P*adj < 0.001); *Scatomorpha sp900545405* was more abundant postchallenge (*P*adj = 0.020) (Supplementary Figure 4, http://links.lww.com/AJG/D912; Figure 5). Metatranscriptomic determinants included lower triacylglycerol and PUFA synthesis expression (*P*adj < 0.001 and *P*adj = 0.001) and greater postchallenge increase in GABA degradation (*P*adj = 0.041) (Supplementary Figure 5, http://links.lww.com/AJG/D912; Figure 5). No significant differences were detected for GOS degradation (Supplementary Figure 6, http://links.lww.com/AJG/D912).

**Figure 5.**
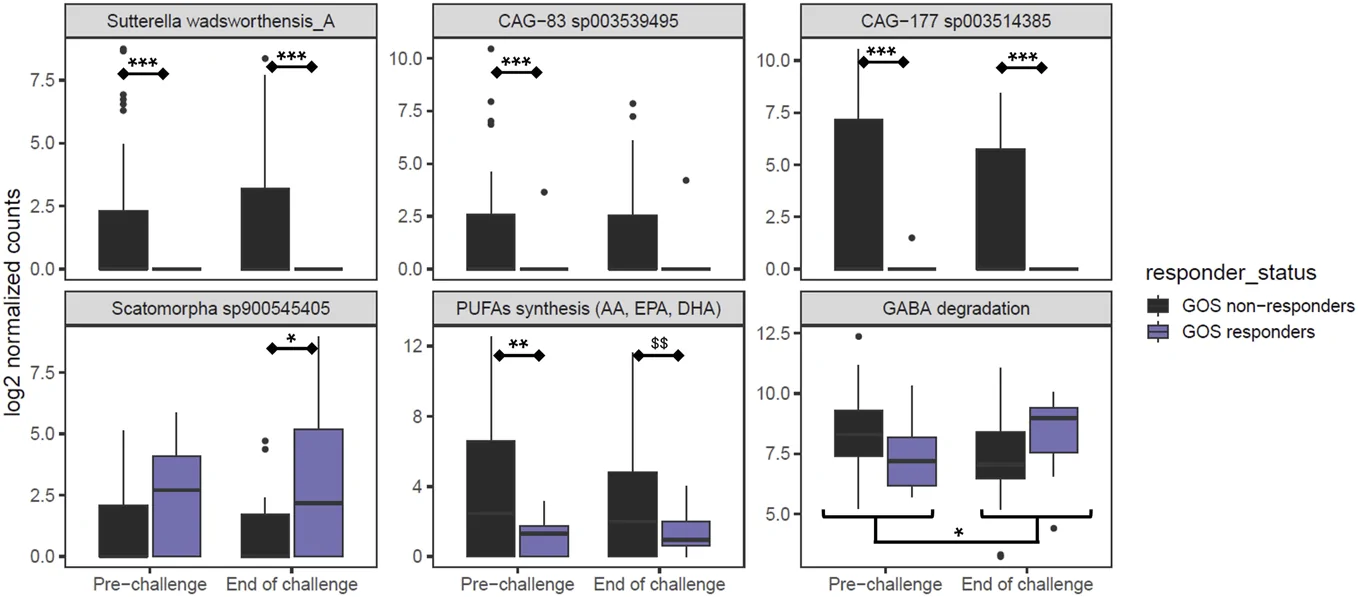
Boxplots of significant mOTUs (metagenomic) and GBMs (metatranscriptomic) by GOS responder status. ***Padj < 0.001, **Padj < 0.01, *Padj < 0.05, $$*P* < 0.01 with DESeq2 analysis.

No prechallenge fermentation differences were observed. Postchallenge, fructan responders had higher fecal iso-butyrate (*P* = 0.015) and total branched-chain fatty acids (BCFA) (*P* = 0.033) concentrations, driven by an increase in iso-butyrate during the challenge (*P* = 0.019) (Figure 6; Supplementary Table 3, http://links.lww.com/AJG/D912).

**Figure 6.**
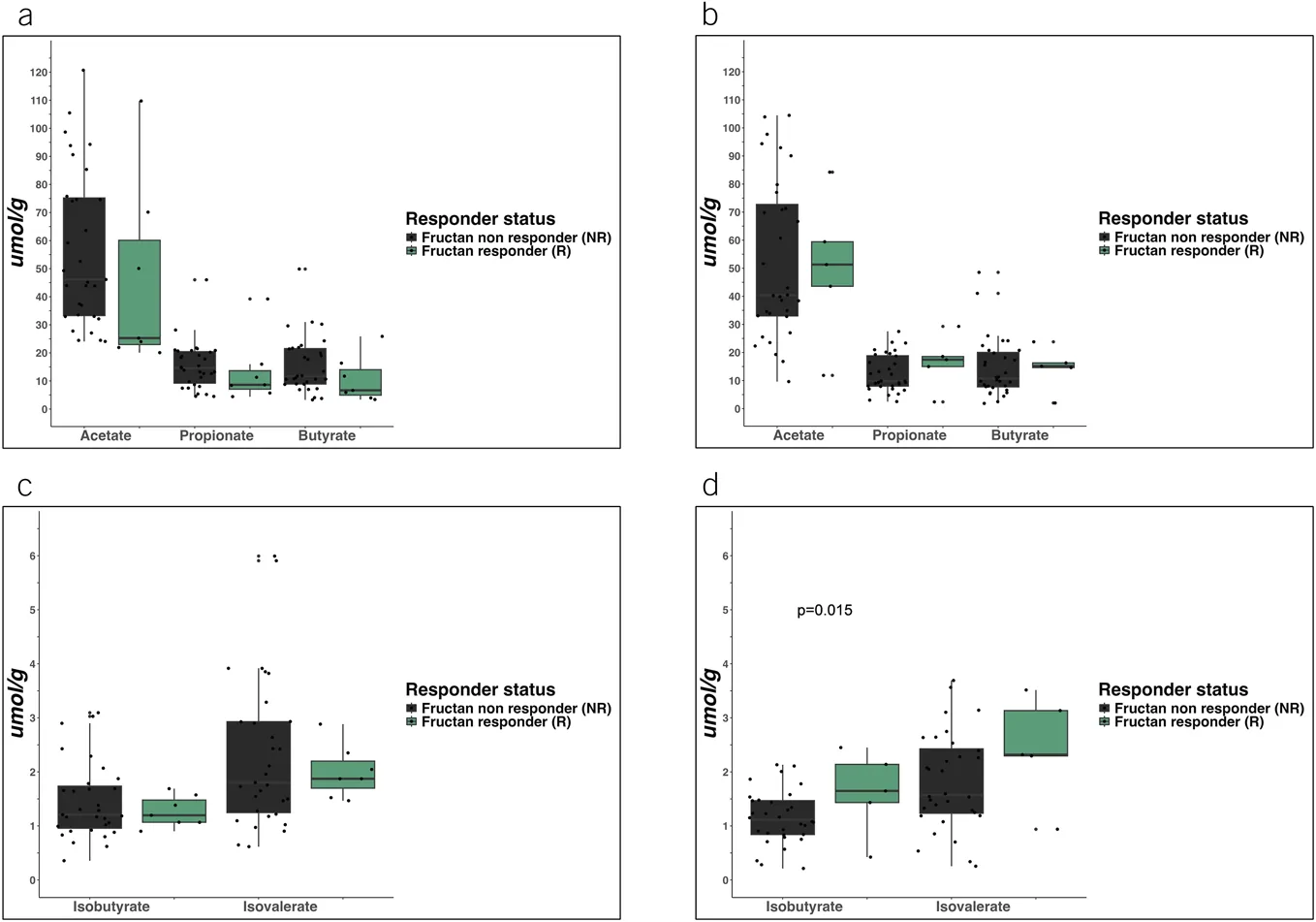
Boxplots of fecal SCFA and BCFA at prechallenge and end of challenge by fructan responder status. Assessed with Mann-Whitney *U* test (prechallenge) and ANCOVA (end of challenge). (**a**) fecal SCFA prefructan challenge; (**b**) fecal SCFA end of fructan challenge; (**c**) fecal BCFA prefructan challenge; (**d**) fecal BCFA end of fructan challenge.

Breath H_2_ was higher prechallenge in GOS responders (*P* = 0.011) (Figure 7), but this difference disappeared postchallenge due to a reduction in H_2_ production (*P* = 0.008). No other breath fermentation differences were observed. Fermentation determinants and mechanisms are presented in Table 4, with delta values in Supplementary Table 3 (http://links.lww.com/AJG/D912). Within the VOC analysis, few differences were found; p-cresol was higher prechallenge in stool from fructan responders (*P* = 0.009) (Supplementary Figure 8, http://links.lww.com/AJG/D912).

**Figure 7.**
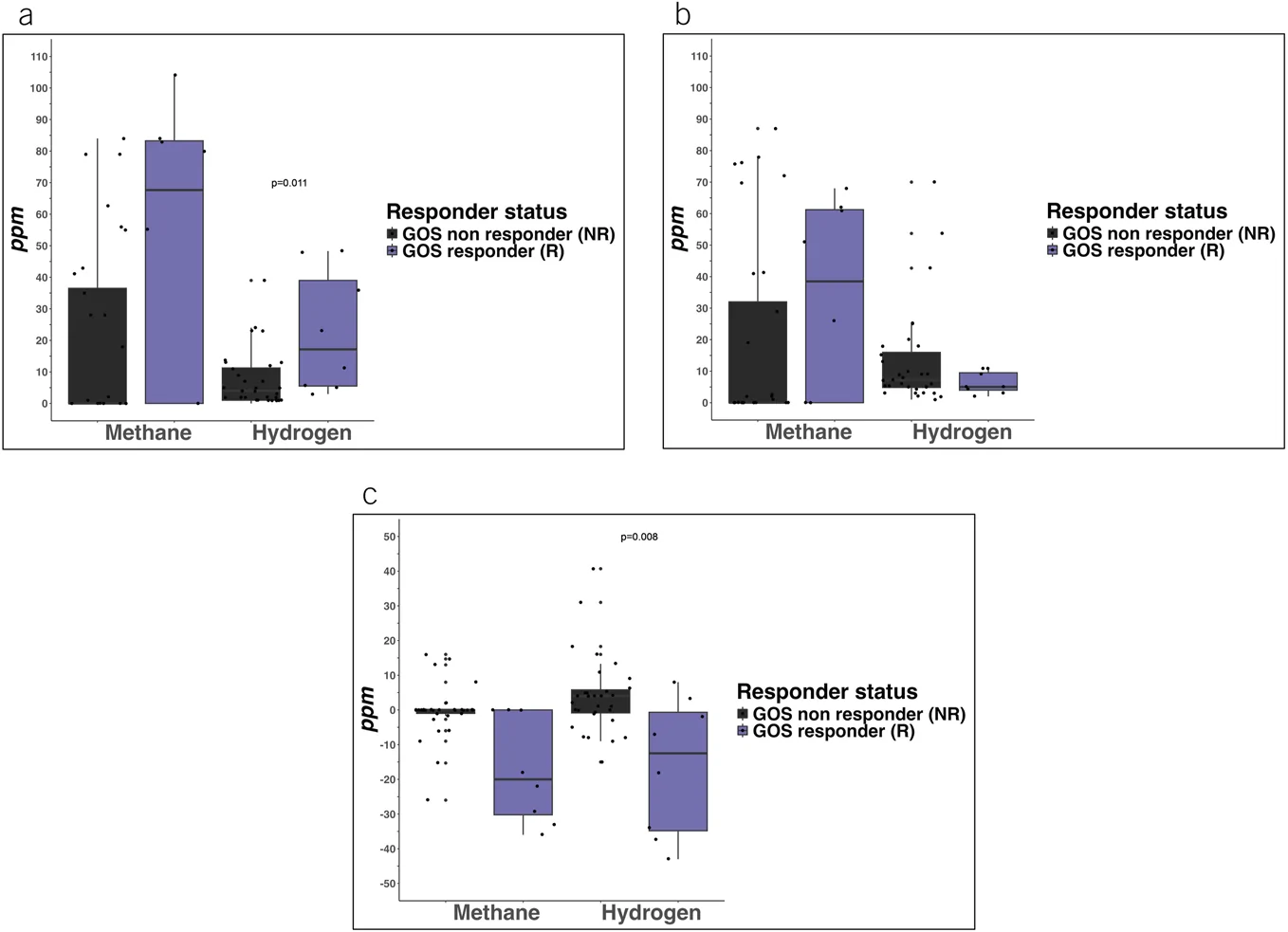
Boxplots of breath methane (CH_4_) and hydrogen (H_2_) at prechallenge, end of challenge and delta between timepoints by GOS responder status. Assessed with *U* test (prechallenge) and ANCOVA (end of challenge). (**a**) Breath gas (CH_4_ and H_2_) pre GOS challenge; (**b**) breath gas (CH_4_ and H_2_) end of GOS challenge; (**c**) breath gas (CH_4_ and H_2_) delta between pre and end of GOS challenge values.

**Table 4.**
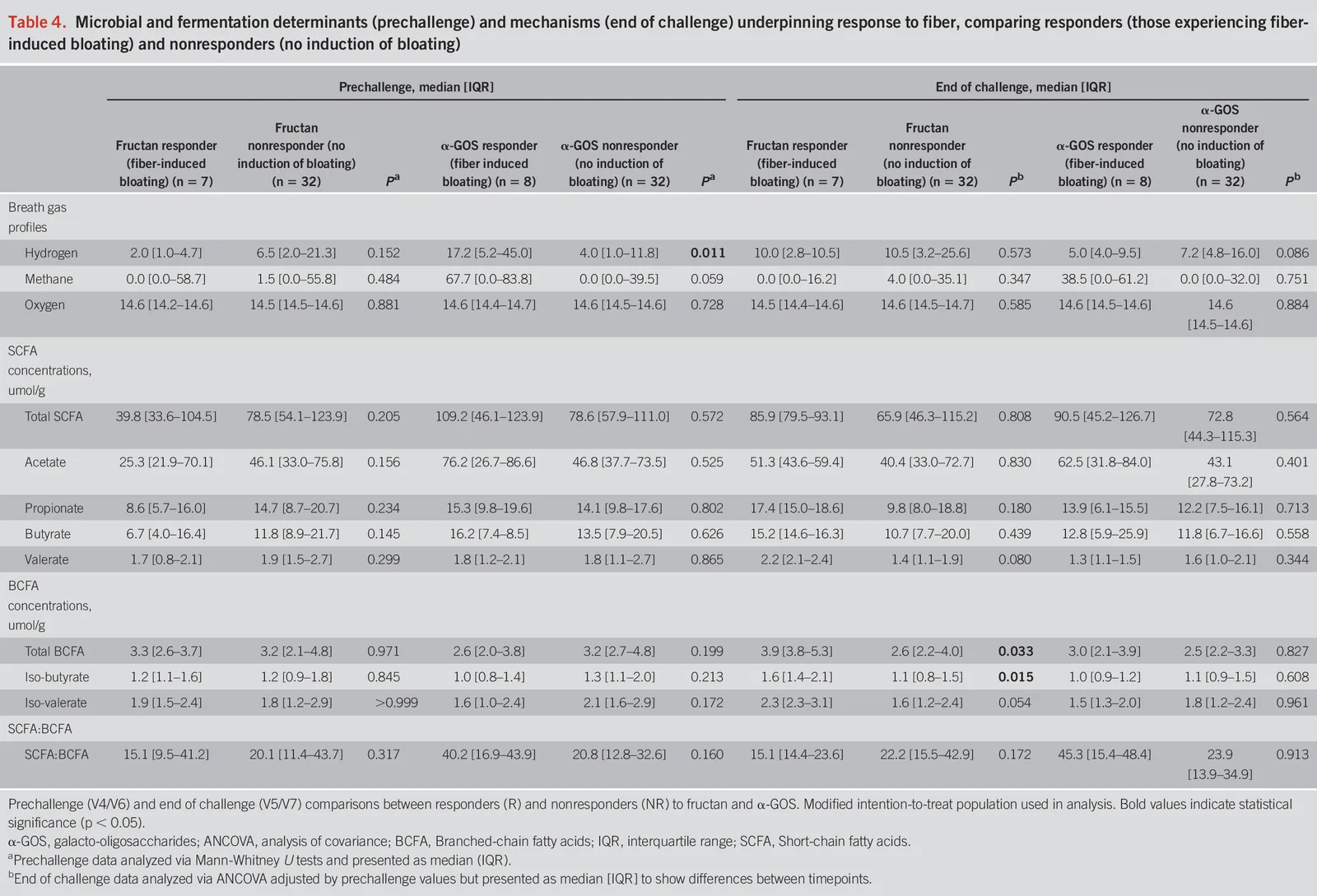
Microbial and fermentation determinants (prechallenge) and mechanisms (end of challenge) underpinning response to fiber, comparing responders (those experiencing fiber-induced bloating) and nonresponders (no induction of bloating)

### Clinical, nutritional, microbial, and fermentation predictors of visceral hypersensitivity

Among the 22 participants undergoing barostat assessment (mPP population), 11 (50%) were classified as having VH. VH status was unrelated to responder status, and baseline clinical differences were minimal. VH participants reported greater flatulence (IGQ, *P* = 0.045) and higher hunger scores (*P* = 0.038). Only zinc intake differed nutritionally, being higher in non-VH participants (*P* = 0.037). No differences were found in breath gases or fecal SCFAs. Microbially, 12 CAZymes, including 9 glycoside hydrolases (GH27), one polysaccharide lyase, one carbohydrate-binding module, and one glycosyltransferase, were more highly expressed in VH participants (Supplementary Tables 4–7, http://links.lww.com/AJG/D912).

## DISCUSSION

The aim of this study was to identify individuals with functional bloating who experience induction of bloating to fructans or GOS (responders), fibers known to elicit food-induced bloating, and to characterize the clinical, microbial, and dietary factors underpinning these responses.

Our primary outcome was bloating response to the fibers, with “responders” (those experiencing fiber-related induction of bloating) defined as participants not having adequate relief of bloating on at least 2 of the last 3 days of the fiber challenge period. Only a small proportion of participants met responder criteria (17.9% for fructan, 20% for GOS) despite recruitment targeting that self-reporting symptom response to these fibers. The strict criteria nonetheless generated distinct responder and nonresponder groups, well differentiated by bloating and abdominal pain scores (GRS, IGQ) and abdominal girth (25). The interventions were well tolerated, indicating that the dosing successfully induced mild-to-moderate, reproducible symptoms without adverse effects.

Before the fructan challenge, responders showed higher microbial alpha diversity, which decreased postchallenge to levels similar to nonresponders. CAZyme diversity, however, remained higher, suggesting potentially greater overall carbohydrate-active enzymatic capacity among fructan responders. The lack of difference in fructan degradation gene expression (e.g., GH32) suggests that enzymatic potential may not be driven by global pathways but may depend on species-level functions influencing downstream carbohydrate metabolism.

Increased expression of GABA degradation genes in responders suggests potential involvement of GABA pathways. Given that GABA receptor signaling attenuates visceral pain (26) and exogenous GABA reduces hypersensitivity (27), greater GABA breakdown might reduce receptor activity, heightening visceral sensitivity. However, no baseline link between GABA degradation and visceral hypersensitivity was found, and prospective studies are needed to clarify GABA's role in food-induced symptoms.

A low *Faecalibacterium*/*Bacteroides* ratio predicted response to both fibers, consistent with findings linking this imbalance to altered gut ecology among Bacteroides-dominant individuals (28). Although our study population was not Bacteroides-dominant, this observation highlights the potential role of microbial community structure in food-induced gut symptoms.

Fermentation outcomes revealed nuanced responses. Fructan responders had higher fecal isobutyrate and total BCFA after challenge, suggesting a shift from carbohydrate to proteolytic fermentation. In contrast, GOS responders displayed higher baseline breath H_2_ but a marked reduction when tested after the 7-day challenge period, abolishing group differences postintervention. This was an interesting finding as our objective was not to measure gas changes to an acute fiber challenge, but rather to measure background breath gas when consuming low(er) levels of the fiber and then again after 7 days of a challenge with a specific fiber to identify whether breath gas composition was different in those who go on to respond and also to investigate whether the composition of breath gas changes differentially on chronic (rather than acute) exposure to the fiber. Implementing this approach, we have found patterns and changes that could be reflective of fluctuations in background breath gas in individuals susceptible to fiber-induced bloating. The reduction in H_2_ in GOS responders may reflect dietary adjustment as they reported reduced fiber and FODMAP intake, limiting fermentable substrate delivery to the colon and lowering H_2_ excretion. Alternatively, greater intraluminal H_2_ retention rather than excessive production could underlie symptom onset. The concomitant increase in abdominal girth among responders supports this, consistent with reports linking gas retention and bloating in only a subset of patients (29). More broadly, altered processing of intestinal stimuli and abnormal visceromotor reflexes, rather than gas volume alone, likely contribute to distension (9). Given the multifactorial nature of bloating and the absence of a clear role for visceral hypersensitivity in this study population, causal links between gas dynamics and symptom generation remain speculative.

Our VOC analysis identified higher stool p-cresol concentrations as a determinant of fructan response. P-cresol, a uremic toxin produced through microbial fermentation of tyrosine (30,31), and associated with proteolytic bacteria (32), exerts genotoxic effects *in vitro* (33), and increases with high meat intake and proteolytic bacterial proliferation (34). This pattern, elevated p-cresol and BCFA despite abundant fermentable carbohydrate, may indicate a shift toward proteolytic fermentation and impaired carbohydrate metabolism, reflecting broader microbial dysregulation in food-induced bloating.

Strengths of this study include the randomized crossover design, which allowed reducing sample size versus parallel trials. The inclusion of a placebo challenge during screening effectively excluded nocebo responders (35). Visceral hypersensitivity was assessed via validated barostat measurements, and use of both metagenomic and metatranscriptomic analyses provided complementary insights into microbial presence and expression.

Limitations include the small number of responders (7 fructan, 8 GOS), thus limiting statistical power. The overall pool of study participants may also be a contributing factor to the small number of responders and thus limiting statistical power, with a greater starting study population potentially overcoming the low percentage of responders defined. The GMM database, though curated, is not exhaustive and may not capture the full metabolic landscape. Moreover, stool-based metatranscriptomics may not fully reflect colonic metabolic activity or spatial variation. Finally, although the placebo challenge during screening was a strength, in some individuals glucose may reach the colon and undergo fermentation, potentially eliciting gastrointestinal symptoms, which may have excluded some individuals who were not nocebo responders (36).

In conclusion, this study identified individuals with functional bloating who experience symptom induction to fructans and GOS. Greater baseline GI symptom burden and abdominal girth were associated with more pronounced clinical responses. Distinct microbial and metabolic signatures, including higher baseline breath H_2_ (GOS), a higher baseline microbial diversity reduced by fermentable fiber exposure (fructan), and increased CAZyme activity not specific to fructan degradation, suggest complex microbial contributions to symptom generation. Abdominal girth emerged as a key clinical marker, underscoring the interplay between distension, gas retention, and visceromotor responses. Overall, these findings advance understanding of predictors and mechanisms of fiber-induced bloating and emphasize the importance of dietary and microbial factors in personalizing dietary interventions for functional gut disorders.

## CONFLICTS OF INTEREST

**Guarantor of the article** Kevin Whelan, PhD.

**Specific author contributions:** D.N.F. (Data curation, Formal analysis, Visualization, Writing – original draft). A.C. (Conceptualization, Data curation, Formal analysis, Methodology, Visualization, Writing – review & editing). B.W. (Investigation, Methodology, Project administration, Writing – review & editing). D.S. (Data curation, Formal analysis, Project administration, Writing – review & editing). P.S.G. (Investigation, Project administration, Writing – review & editing). R.S. (Investigation, Writing – review & editing). C.P. (Investigation, Writing – review & editing). S.M. (Investigation, Writing – review & editing). S.M.S. (Investigation, Supervision, Writing – review & editing). L.Q. (Conceptualization, Formal analysis, Methodology, Writing – review & editing). M.P. (Data curation, Formal analysis, Methodology – review & editing). S.S. (Validation, Writing – review & editing). J.T. (Conceptualization, Formal analysis, Methodology, Supervision, Writing – review & editing). B.L.N. (Conceptualization, Formal analysis, Methodology, Project administration, Supervision, Writing – review & editing). M.R. (Conceptualization, Funding acquisition, Methodology, Project administration, Supervision, Writing – review & editing). K.W. (Conceptualization, Formal analysis, Funding acquisition, Methodology, Project administration, Supervision, Writing – original draft).

**Financial support:** This study was exclusively funded by Danone Research & Innovation.

**Potential competing interests:** D.N.F., P.S.G., D.S., R.S., C.P. and S.M. have no interests to declare. A.C., L.Q., M.P., S.S. and B.L.N. are employees of Danone and J.T. was an employee of Danone at the time the research was conducted. M.S. has received honoraria from Coloplast as a member of their Advisory Board and from Laborie for contribution to their Education Program. B.W. has received research and consultancy funding from Medtrition Inc and is the holder of a joint patent to use volatile organic compounds in the diagnosis and dietary management of irritable bowel syndrome. M.R. has received research grants from commercial funders including Almond Board of California, Danone, and International Nut and Dried Fruit Council, is the co-founder of food brand Bio&Me and is the holder of a joint patent to use volatile organic compounds in the diagnosis and dietary management of irritable bowel syndrome. K.W. has received research grants from commercial funders including Almond Board of California, Danone, and International Nut and Dried Fruit Council and has received speaker fees from Yakult UK and Danone, and is the holder of a joint patent to use volatile organic compounds in the diagnosis and dietary management of irritable bowel syndrome.

**Data sharing statement:** Data described in the manuscript are not publicly available due to ethical and participant confidentiality restrictions, and because consent for data sharing was not obtained. Accordingly, the data cannot be shared.

**Clinical Trials Registration:** NCT04802798.

**Ethical statement:** Ethical approval was received from the Research Ethics Committee, King's College London (Reference: HR-19/20-18704). All participants provided informed consent before participation.

## Supplementary Material

## ABBREVIATIONS:

ANCOVA: analysis of covariance
ANOVA: analysis of variance
BCFA: branch-chain fatty acids
BSFS: Bristol Stool Form Scale
CAZyme: carbohydrate active enzyme
DGBI: disorder of gut-brain interaction
DP: degree of polymerization
FODMAP: fermentable oligosaccharides
disaccharides: monosaccharides and polyols
GABA: gamma-aminobutyric acid
GH: glycoside hydrolase
GI: gastrointestinal
GMM: gut metabolic module
GOS: α-galacto-oligosaccharides
GSQ: Global Symptom Questionnaire
GSRS: Gastrointestinal Symptoms Rating Scale
IGQ: Intestinal Gas Questionnaire
ITT: intention to treat
mITT: modified intention to treat
mOTUs: metagenomic operational taxonomic units
*P*adj: adjusted *P*-value
PERMANOVA: permutational multivariate analysis of variance
SCFA: short-chain fatty acids
VH: visceral hypersensitivity
VOC: volatile organic compounds
