# Supplementary material for: Gut Microbiome Composition and Function, Diet, and Clinical Factors in Relation to Fermentable Carbohydrate-Induced Bloating: A Double-Blind, Randomized, Crossover Trial
Source: Am J Gastroenterol. 2026 Mar 17;121(11):2695–709. doi: 10.14309/ajg.0000000000003997 (PMC13722433; doi:10.14309/ajg.0000000000003997)
Supplement: Supplementary file 3 [file acg-121-2695-s003.docx]

**Gut Microbiome Composition and Function, Diet and Clinical Factors in Relation to Fermentable Carbohydrate-Induced Bloating: A Double-Blind, Randomized, Crossover Trial**

**Supplementary Material**

**Supplementary Methods**

**Recruitment strategies**

Participants were recruited via several approaches including circular emails to the university population of King’s College London. Participants were also recruited via advertising in London newspapers and magazines, classified websites, online clinical trial databases, social media, and via recruitment posters and leaflets placed in institutions.

**Inclusion and exclusion criteria**

Inclusion criteria comprised participants aged 18-60 years with BMI 19-29 kg/m^2^, who fulfilled Rome IV Criteria for functional bloating [1], but not for IBS, functional constipation or functional diarrhea, and had not been diagnosed with any other chronic GI disease including inflammatory bowel disease or celiac disease. We recruited participants who reported heightened response to foods predominantly high in just one of fructans or α-GOS, but who due to avoidance of perceived trigger foods had well-controlled symptoms. The objective was to recruit equal proportions of responders to fructans and α-GOS.

Exclusion criteria included excessive habitual intake of fructans, α-GOS or polyols, fructose, as defined by a clinician as the sole cause of symptoms; individuals with specific dietary requirements (e.g. strict low Fermentable Oligosaccharides, Disaccharides, Monosaccharides and Polyols (FODMAP) diet); participation in a clinical trial within the previous 4 weeks; known or suspected allergy to any component of study product(s). Full eligibility criteria are provided in Supplementary Materials. receiving treatment for anorexia, weight loss or any form of treatment likely to interfere with metabolism or dietary habits; antibiotics or proton pump inhibitor use within the previous 2 months; current use of medication with potential central nervous system effects; individuals with previous digestive surgery (except for appendectomy and cholecystectomy performed more than 2 years ago); athletes, defined as performing strenuous daily exercise for more than 1.5 hours at a time; oral disease that may impact on breath sampling; individuals starting or routinely taking drugs that might modify GI function; women who reported they were pregnant, lactating or planning pregnancy; recent and/or ongoing consumption of probiotic/prebiotics supplements as well as anticipated changes to consumption of naturally probiotic/prebiotic containing foods; anticipated changes to smoking habits in the next 2 months; ongoing abuse of alcohol (> 40 units/week), non-prescription drugs or any other medication. In addition, participants who experienced moderate or severe bloating to the placebo challenge at screening (further details below) were also excluded.

**Fiber supplements**

The supplements were prepared in plain, identical sachets specifically for this study, with only a code and instructions written on the sachet. The supplements were either 8 g/d fructan in 9g of Orafti P95 powder (93% oligofructose), or 8 g/d α-GOS in 9g of oligosaccharide powder (95% plant-derived α-GOS), taken in two doses (first and last meal of the day). Oligofructose (Orafti® P95, Beneo, Germany) (fructan intervention) was derived from enzymatic hydrolysis of chicory inulin and consisted of oligosaccharides composed of fructose units linked together by ß-(2,1)-linkages. The product used was 97% dry matter containing a minimum of 93.2% oligofructose. α-galacto-oligosaccharide (AlphaGOS® 95 Pea, Olygose SAS, France) (α-GOS intervention) was derived from yellow peas (*Pisum sativum*) and consisted of 95% of α(1-6)-linked alpha-galacto-oligosaccharides. The product used had at least 95% dry matter.

The intervention products were presented as white powder dissolvable in water and had similar texture, color, taste, packaging and nutritional content to preserve the double-blind methodology. Participants were instructed to mix the product with 150mls of water using a shaker and then to rinse the shaker with a further 150mls water and drink this to ensure they consume the full dose.

Our rationale for the intervention doses (8 g/day) was based on previous evidence that shows that doses of ≤6 g/day fermentable fibers may actually improve or have no impact on symptoms [2-7], whereas doses of ≥10 g/day showed a substantial increase in GI symptoms in comparison to control [8-10]. We therefore aimed to find an effective balance between these dosages, which based on our observations, have been justified.

**Placebo challenge**

The study included a placebo challenge as part of the screening process to remove participants with a nocebo response to the inert challenge. Participants received an initial single-blinded placebo (8g/day glucose) challenge for 7-days. Glucose was used as placebo as it is easily absorbed in the small intestine and would contribute minimally to colonic fermentation and subsequent bloating. Those who responded symptomatically to this (nocebo responders) were excluded from further participation. The placebo challenge can be found in Figure 1 pertaining to Visit 1 and Visit 2.

**Randomization and blinding**

A computerized randomization with blocks of 4, stratified for both gender and reported fiber sensitivity (fructan or α-GOS), was prepared by an independent researcher not otherwise involved in the trial.

After the exclusion of placebo challenge responders, each participant was randomly allocated to either a fructan/ α-GOS sequence or a α-GOS/fructan sequence. Participants in the fructan/α-GOS sequence received the fructan challenge during the first period and the α-GOS challenge during the second period. Participants were then blinded to all three interventions (two fiber challenges and placebo challenge) and investigators and staff were blinded to the sequence of the two fiber challenges. The sequence of challenges was determined using the clinical trial randomization website sealedenvelope.com. Supplements were provided to participants in identical boxes labelled with randomization number, followed by 1 (placebo challenge), 2 and 3 (fructan/α-GOS) by the independent researcher.

**Visceral hypersensitivity**

Visceral hypersensitivity was determined using a standardized technique employing an electromechanical barostat, performed by experienced clinical practitioners at Queen Mary University London. Following a 12 hour overnight fast, participants underwent a 500 ml tap water enema at body temperature. Assessment of rectal sensitivity and compliance was then performed using a 600 ml non-compliant polyolefin bag (Mui Scientific; Mississauga, Canada) attached to a barostat (Distender II; G&J Electronic inc. Toronto, Canada). With the subject in the left lateral position, the barostat bag was inserted into the rectum, and following a conditioning distension and evaluation of minimum distending pressure, rectal pressures and volumes were recorded using a stepwise ascending method of limits protocol (4 – 60 mmHg in 4mmHg steps, or to maximum toleration). Pressure-volume curves were constructed in Excel (Microsoft, USA) post procedure, with rectal compliance (slope [δV/δP] of the linear part of the sigmoidal compliance curve) calculated. Normal compliance was taken as 8.6–19.1 mls / mmHg (unit reference values). Immediately prior to the start of the procedure, level of stress was assessed in the study subject using a visual analogue score anchored at 0 (“not stressed”) to 10 (“extremely stressed”). All pre-menopausal female participants were studied between days 6 and 24 of their menstrual cycle, or while taking contraceptive medication. Alcohol- and caffeine-containing products were prohibited for 24 hrs before the study. Rectal hypersensitivity was diagnosed if pain threshold was <32 mmHg [11].

**Metagenomic and metatranscriptomics**

*Sample collection*

A total of 396 fecal samples were collected in the study (198 for metagenomic analysis and 198 for metatranscriptomic analysis) from 41 subjects at up to 5 time points. Fresh fecal samples were collected using DNA/RNA Shield™ Zymo tubes and stored on ice for less than 1 hour before being aliquoted and stored at −80°C.

*DNA and RNA extraction*

All samples were then processed for DNA and RNA extraction before metagenomic and metatranscriptomic profiling. DNA and RNA extraction were performed using ZymoBIOMICS MagBead DNA (D4302) and ZymoBIOMICS MagBead RNA (R2137/8) kits, respectively. The RNA extraction was followed by rRNA depletion using the Ribo-Zero Plus kit (Illumina).

*Sequencing and bio-informatic post-processing*

Paired-end sequence reads were generated using the Illumina NovaSeq 6000 system. FASTQ read sequence files were generated using bcl2fastq version 2.20 (Illumina). Initial quality assessment was based on data passing the Illumina Chastity filtering. Subsequently, reads containing PhiX control signal were removed using an inhouse filtering protocol. In addition, reads containing (partial) adapters were clipped (up to a minimum read length of 50 bp). Sequencing data were then quality-controlled using fastp [12] and the sequences that align to the human genome discarded.

For metagenomic data, metagenomic operational taxonomic units (mOTUs) were searched and quantified on the remaining sequences using mOTUs v3.0.3 [13]. Taxonomic annotation was performed via GTDB v2.1 on database release 207: <https://github.com/motu-tool/mOTUs/wiki/GTDB-taxonomy-for-the-mOTUs>. After rarefaction at 5,000 mOTUs-mapped sequences, the number of mOTUs, Shannon diversity and Bray-Curtis dissimilarity were computed using the vegan R package. In addition, the Aitchison distance was computed on unrarefied data after adding a pseudo-count of 1 using the coda.base R package. Taxonomic data at the genus level were generated by aggregation.

For both metagenomic and metatranscriptomic data, genes were searched and quantified on the remaining sequences using bwa against the Integrated Gene Catalog [14] and counts were scaled by gene length. Gut Metabolic Modules (GMMs, v1.0.7, <https://github.com/raeslab/GMMs>) and Gut Brain Modules (GBMs, <https://raeslab.org/software/gbms.html>) counts were estimated using the omixerRpm R package (v0.3.2) and carbohydrate-active enzymes (CAZymes) aggregated counts were computed. For metagenomics, after rarefaction at 10,000,000 reads, gene richness, the number of CAzymes and Shannon diversity on CAzymes were computed using the vegan R package.

**Volatile organic compounds**

Volatile organic compound (VOC) analysis was performed using gas-chromatography mass-spectrometry (GC-MS) on a PerkinElmer Clarus 500 GC-MS quadrupole benchtop system (Beaconsfield, UK) and Combi PAL auto-sampler (CTC Analytics, Switzerland) for all stool samples collected at baseline and week-4. Prior to GC-MS analysis VOCs were extracted from the headspace of 450-500 mg of stool in 10 ml headspace vials (Sigma-Aldrich, Dorset, UK) using solid phase micro-extraction (SPME). A SPME fiber coating of DVB-CAR-PDMS 50/30 µm (1 cm) (Sigma-Aldrich, Dorset, UK) was used. The optimized SPME extraction method and GC-MS conditions were the same as previously published by Reade et al [15]. Individual VOCs had to be present in at least 50% of samples in at least one experimental condition to remain in the analysis.

**Statistical analysis**

*Sample size*

Pre-FIBRe was an exploratory, hypothesis generating study, and as such there were no available data to estimate the expected differences in microbiome profiles between responders and non-responders to fructan or α-GOS to inform a sample size calculation. However, we used screening questionnaires to recruit equal numbers self-reporting “responders” to fructan containing foods (wheat, onion, garlic) and α-GOS (beans, legumes), anticipating a 50% response rate to fructan, in line with a previous carbohydrate challenge study by our group [16]. Based on previous trials where carbohydrate challenges have been given to participants with DGBIs, with sample sizes 24-32 participants [16, 17], a total of 30 participants was targeted for recruitment, and assuming a 20% drop-out rate we would need 40 randomized participants to participate in this trial. With a pooled placebo response rate of 45% in DGBIs [18], reflecting the numbers with a nocebo response during the placebo screening phase, the number of participants needed to enter the placebo screening phase was 72.

*Analysis sets*

We used the modified intention to treat (mITT) population, which consisted of all participants who were consented, randomized and completed at least one challenge (at least 3 days of data were provided). Completion of a challenge was defined as consuming 7 days of the fiber or consuming the fiber for at least 3 days but needing to cease early due to excessive symptoms – in both cases, the last 3-days of their diary was used for the analysis of study outcomes. Where a participant only completed one challenge and withdrew before completing the second challenge, their data was used in the mITT analysis for the challenge they completed, but not for the challenge they did not complete.

To analyze predictors of visceral hypersensitivity (VH), we used a modified per protocol population (mPP) which comprises the mITT population, excluding participants who only completed one challenge, had a protocol deviation (undertaking activities contradicting inclusion criteria (e.g., consumption of antibiotics), whose washout period was ≥ 28 days, and as this analysis was focused on VH metrics, it also excludes all participants who did not complete the barostat study prior to the end of the study (V7).

*Data checks*

Prior to statistical analysis, data checks were performed. Pre-challenge data was used as the ‘baseline’ time point to address the determinant and mechanism research objectives. As such, data from V4 and V6 was pooled for each fiber challenge for comparisons, to check for order effects, assuming equivalence at the two time points. The equivalence between V4 and V6 for study outcomes (clinical, nutritional and fermentation profiles) was assessed using paired t-tests or Wilcoxon signed-rank tests depending on data distribution for most parameters, stratified permutational analyses of variance (PERMANOVA) for beta-diversity indices and DESeq2 models with subject and responder status effects [19] for mOTUs. This analysis found one minor difference in clinical profiles across the pre-challenge data, belching, which was higher at V4 compared to V6 according to both the IGQ (p = 0.006) and GSRS (p = 0.003). The only difference in nutritional profile was selenium, which was marginally higher at V4 compared to V6 (p = 0.049). Order effects were also assessed to verify that the response to challenges was independent of the order and period. Responses to the challenges across the two randomized sequences (fructan-α-GOS or α-GOS-fructan) was assessed using Chi-squared tests and Fisher’s Exact tests and found to have no order effects (p = 0.417 and p = 0.336, respectively).

*Visceral Hypersensitivity*

Analysis of clinical, nutritional and fermentation predictors of VH was performed on data at trial baseline (V3) to avoid the need for this invasive test at each pre-challenge visit (V4 and V6). Unpaired t-tests or Mann-Whitney tests were performed to compare differences between participants with visceral hypersensitivity (VH) and without (non-VH).

**Gut Microbiome Composition and Function, Diet and Clinical Factors in Relation to Fermentable Carbohydrate-Induced Bloating: A Double-Blind, Randomized, Crossover Trial**

**Supplementary Results**

| **Supplementary Table 1. Clinical mechanisms (delta pre- to end of challenge) underpinning response to fiber** | | | | | | |
| --- | --- | --- | --- | --- | --- | --- |
| **Variable** | **Fructan responder delta**  **(fiber-induced bloating)** | **Fructan non-responder delta**  **(no induction of bloating)** | **P** | α-**GOS responder delta**  **(fiber-induced bloating)** | α-**GOS non-responder delta**  **(no induction of bloating)** | **P** |
| **IGQ** | | | | | | |
| Bloating | +24.2 [5.8, 47.5] | +3.8 [-1.7, 15.6] | **0.007** | +22.1 [9.0, 34.8] | +0.4 [-1.7, 12.9] | **0.011** |
| Flatulence | +15.0 [0.0, 43.3] | +3.3 [-2.5, 18.3] | 0.223 | +12.5 [-9.2, 47.1] | +3.3 [-1.2, 12.9] | 0.623 |
| Belching | +22.5 [0.0, 30.0] | 0.0 [-3.7, 5.0] | 0.056 | 0.0 [0.0, 11.2] | 0.0 [-3.7, 8.7] | 0.760 |
| Bad breath | 0.0 [0.0, 10.0] | 0.0 [0.0, 4.4] | 0.768 | 0.0 [0.0, 0.0] | 0.0 [-5.0, 5.0] | 0.911 |
| Stomach rumbling | +5.0 [0.0, 10.0] | 0.0 [-5.0, 8.7] | 0.355 | 0.0 [0.0, 21.9] | 0.0 [0.0, 6.9] | 0.687 |
| Difficult gas evacuation | +12.5 [0.0, 37.5] | 0.0 [0.0, 12.5] | 0.062 | +12.5 [3.1, 37.5] | 0.0 [0.0, 9.4] | **0.013** |
| Global score | +13.9 [3.3, 19.7] | +2.2 [-1.8, 9.2] | **0.041** | +9.1 [1.1, 17.1] | +1.5 [-2.4, 8.8] | 0.088 |
| Global score (except bloating) | +13.8 [1.5, 15.7] | +1.7 [-1.0, 8.5] | 0.095 | +8.5 [-0.4, 14.2] | +1.7 [-2.3, 6.6] | 0.171 |
| **GSRS** | | | | | | |
| Pain | 0.0 [0.0, 0.3] | 0.0 [0.0, 0.0] | 0.128 | 0.0 [-0.2, 0.7] | 0.0 [0.0, 0.0] | 0.093 |
| Discomfort | +0.7 [0.0, 1.0] | 0.0 [-0.1, 0.3] | 0.036 | +0.2 [-0.2, 1.22] | 0.0 [0.0, 0.3] | 0.447 |
| Heartburn | 0.0 [0.0, 0.0] | 0.0 [0.0, 0.0] | >0.999 | 0.0 [0.0, 0.2] | 0.0 [0.0, 0.0] | 0.372 |
| Reflux | 0.0 [0.0, 0.0] | 0.0 [0.0, 0.0] | 0.967 | 0.0 [0.0, 0.0] | 0.0 [0.0, 0.0] | 0.551 |
| Nausea | 0.0 [0.0, 0.0] | 0.0 [0.0, 0.0] | 0.413 | 0.0 [0.0, 0.2] | 0.0 [0.0, 0.0] | >0.999 |
| Gurgling | 0.0 [0.0, 0.7] | 0.0 [0.0, 0.3] | 0.576 | 0.0 [0.0, 0.6] | 0.0 [-0.2, 0.0] | 0.333 |
| Bloating | +0.7 [0.5, 1.7] | 0.0 [-0.1, 0.6] | **0.002** | +1.0 [0.5, -0.2] | +0.2 [0.0, -0.3] | **0.049** |
| Belching | -0.7 [0.0, 1.0] | 0.0 [0.0, 0.3] | 0.052 | 0.0 [-0.3, 0.5] | 0.0 [-0.2, 0.2] | 0.841 |
| Flatulence | +0.7 [0.0, 1.3] | +0.3 [0.0, 0.6] | 0.079 | +0.3 [-0.2 0.7] | 0.0 [0.0, 0.3] | 0.275 |
| Constipation | 0.0 [0.0, 0.3] | 0.0 [0.0, 0.0] | 0.389 | 0.0 [0.0, 0.3] | 0.0 [0.0, 0.0] | 0.194 |
| Diarrhea | 0.0 [0.0, 0.0] | 0.0 [0.0, 0.0] | 0.331 | 0.0 [0.0, 0.0] | 0.0 [0.0, 0.0] | 0.970 |
| Loose stools | 0.0 [0.0, 0.5] | 0.0 [0.0, 0.0] | 0.333 | 0.0 [0.0, 0.2] | 0.0 [0.0, 0.0] | 0.383 |
| Hard stools | 0.0 [0.0, 0.3] | 0.0 [0.0, 0.0] | 0.300 | 0.0 [0.0, 0.0] | 0.0 [0.0, 0.0] | >0.990 |
| Urgency | 0.0 [-0.3, 0.0] | 0.0 [0.0, 0.0] | 0.400 | 0.0 [0.0, 0.2] | 0.0 [0.0, 0.0] | 0.494 |
| Incomplete evacuation | +0.3 [0.0, 1.0] | 0.0 [0.0, 0.0] | 0.132 | +0.5 [0.0, 1.0] | 0.0 [0.0, 0.3] | **0.014** |
| Overall symptoms | +0.3 [-0.3, 1.7] | 0.0 [-0.3, 0.3] | 0.056 | +0.8 [0.1, 1.2] | 0.0 [0.0, 0.2] | **0.009** |
| **GSQ** | | | | | | |
| % days with adequate relief | -66.7 [-66.7, -33.3] | 0.0 [0.0, 0.0] | **< 0.001** | -66.7 [-91.7, -41.7] | 0.0 [0.0, 0.0] | **< 0.001** |
| % days with inadequate relief | +66.7 [33.3, 66.7] | 0.0 [0.0, 0.0] | **< 0.001** | +66.7 [41.7, 91.7] | 0.0 [0.0, 0.0] | **< 0.001** |
| **BSFS** | | | | | | |
| Form | +0.3 [-0.5, 0.5] | -0.2 [-0.6, 0.0] | 0.127 | -0.1 [-0.9, 0.4] | 0.0 [-0.3, 0.4] | 0.684 |
| Frequency | +0.3 [-0.3, 0.7] | 0.0 [0.0, 0.3] | 0.551 | 0.0 [-0.6, 0.6] | 0.0 [-0.3, 0.3] | 0.895 |
| % firm | 0.0 [0.0, 28.6] | 0.0 [0.0, 0.0] | 0.493 | 0.0 [0.0, 0.0] | 0.0 [0.0, 0.0] | 0.642 |
| % normal | 0.0 [-35.7, 0.0] | 0.0 [0.0, 0.0] | 0.065 | 0.0 [0.0, 3.7] | 0.0 [-8.0, 0.0] | 0.131 |
| % loose | 0.0 [0.0, 0.0] | 0.0 [0.0, 0.0] | 0.129 | 0.0 [0.0, 0.0] | 0.0 [0.0, 0.0] | 0.300 |
| **Waist circumference** | | | | | | |
| WC, cm | +2.0 [0.33, 2.0] | 0.0 [-0.6, 0.9] | **0.016** | +3.0 [2.0, -1.0] | +0.0 [0.0, -1.0] | 0.198 |
| **Appetite** | | | | | | |
| Hungry | -4.0 [-13.0, 11.0] | -13.0 [-31.0, 16.0] | 0.587 | -15.0 [-23.0, 1.2] | -7.0 [-23.2, 12.0] | 0.672 |
| Full | -6.0 [-16.0, 24.0] | -2.5 [-16.7, 10.0] | 0.993 | +25.5 [14.5, 45.0] | +1.5 [-14.0, 25.5] | 0.108 |
| Satisfied | -3.0 [-15.0, 18.0] | +1.5 [-15.0, 18.7] | 0.679 | +12.5 [6.7, 18.5] | +6.0 [-21.7, 24.0] | 0.194 |
| Eat now? | -8.0 [-16.0, 2.0] | -6.0 [-28.0, 15.0] | 0.621 | -4.0 [-21.0, -32.0] | +14.0 [-3.0, -24.0) | 0.142 |
| Delta between pre-challenge (V4/V6) and end of challenge (V5/V7) values for responders (fiber-induced bloating) and non-responders (no induction of bloating) to fructan and α-GOS.  Modified intention-to-treat population; population used in analysis. Analyzed via Mann Whitney tests and presented as median [interquartile range (IQR)].  α-GOS, alpha-galactooligosaccharides; GSRS, Gastrointestinal Symptom Rating Scale; GSQ, Gastrointestinal Symptom Question; BSFS, Bristol Stool Form Scale; WC, waist circumference | | | | | | |

| **Supplementary Table 2. Nutritional mechanisms (delta pre- to end of challenge) underpinning response to fiber** | | | | | | |
| --- | --- | --- | --- | --- | --- | --- |
| **Variable** | **Fructan responder delta (fiber-induced bloating)** | **Fructan non-responder delta**  **(no induction of bloating)** | **P** | α-**GOS responder delta (fiber-induced bloating)** | α-**GOS non-responder delta**  **(no induction of bloating)** | **P** |
| **Nutritional intake (per day)** | | | | | | |
| Energy, kcal | +81.2 ± 317.6 | +46.9 ± 281.5 | 0.777 | -54.5 ± 445.0 | -11.4 ± 344.5 | 0.767 |
| Protein, g | -2.4 ± 10.5 | +3.4 ± 15.6 | 0.355 | +6.9 ± 28.8 | +3.2 ± 20.7 | 0.677 |
| Fat, g | +3.9 + 21.5 | +4.0 ± 16.5 | 0.629 | +3.4 ± 19.9 | -1.3 ± 22.8 | 0.496 |
| Saturated fat, g | +3.4 ± 10.8 | +1.2 ± 8.2 | 0.782 | -0.1 ± 8.7 | -1.8 ± 8.9 | 0.627 |
| Carbohydrates, g | +29.3 ± 57.6 | -0.9 ± 35.8 | 0.079 | -30.7 ± 61.9 | -1.4 ± 37.1 | 0.091 |
| Starch, g | +5.8 ± 37.3 | -1.9 ± 28.9 | 0.552 | -19.0 ± 46.8 | -1.9 ± 28.3 | 0.191 |
| Sugars, g | +22.1 ± 33.2 | +0.9 ± 22.4 | **0.045** | -12.3 ± 19.4 | -0.4 ± 16.7 | 0.090 |
| Fiber, g | +5.1 ± 4.4 | -0.2 ± 5.1 | **0.014** | -5.2 ± 5.6 | +0.6 ± 5.3 | **0.018** |
| Non-starch polysaccharide, g | +5.6 ± 4.0 | -0.6 ± 3.8 | **<0.001** | -3.6 ± 5.2 | +0.5 ± 3.7 | **0.047** |
| Calcium, g | -96.1 ± 203.3 | +16.1 ± 287.9 | 0.336 | -154.6 ± 315.9 | -17.0 ± 194.5 | 0.309 |
| Iron, mg | +1.2 ± 2.0 | -0.8 ± 4.5 | 0.102 | -1.3 ± 4.1 | +0.03 ± 3.1 | 0.316 |
| Zinc, mg | +0.1 ± 1.2 | -0.3 ± 3.2 | 0.745 | -0.6 ± 3.4 | -0.7 ± 3.5 | 0.939 |
| Sodium, mg | -510.3 ± 557.6 | +279.6 ± 620.0 | **0.004** | -465.8 ± 753.8 | +225.8 ± 798.8 | **0.039** |
| Potassium, mg | +681.5 ± 637.0 | -130.4 ± 654.1 | **0.005** | -58.1 ± 1040.0 | +110.1 ± 473.0 | 0.855 |
| Phosphorus, ug | 74.8 ± 214.7 | -10.9 ± 246.7 | 0.378 | -83.7 ± 371.1 | -16.6 ± 175.0 | 0.454 |
| Magnesium, mg | +69.1 ± 69.6 | -24.6 ± 73.4 | **0.004** | -31.9 ± 85.7 | -2.6 ± 68.1 | 0.306 |
| Iodine, ug | +6.7 ± 47.0 | -2.4 ± 74.3 | 0.758 | +2.65 ± 75.0 | -15.9 ± 89.2 | 0.965 |
| Selenium, ug | -7.5 ± 16.2 | -7.1 ± 33.0 | 0.707 | -5.7 ± 25.7 | -3.0 ± 32.5 | 0.826 |
| Vitamin A, ug | -108.3 ± 758.4 | -13.6 ± 518.5 | 0.690 | -396.1 ± 711.2 | +229.2 ± 489.3 | **0.009** |
| Vitamin C, ug | +35.6 ± 66.6 | -7.5 ± 122.2 | 0.162 | -24.6 ± 40.1 | +2.4 ± 117.2 | 0.174 |
| Vitamin D, ug | -0.03 ± 1.4 | +1.2 ± 9.6 | 0.661 | -0.4 ± 4.4 | +63.4 ± 353.6 | 0.206 |
| Vitamin B9, ug | +54.6 ± 91.3 | -17.5 ± 109.8 | **0.037** | -51.1 ± 129.7 | +21.3 ± 102.7 | 0.099 |
| Vitamin B12, ug | -2.2 ± 3.6 | +2.4 ± 15.3 | 0.153 | -101.0 ± 283.6 | -0.3 ± 3.2 | 0.697 |
| **FODMAP intake, g** | | | | | | |
| Total FODMAPs | -0.2 ± 2.4 | +0.5 ± 4.7 | 0.689 | -2.3 ± 10.7 | +0.8 ± 4.1 | 0.210 |
| Excess fructose | -0.3 ± 0.9 | +0.3 ± 1.2 | 0.258 | -0.6 ± 1.6 | +0.1 ± 1.8 | 0.273 |
| Lactose | +0.05 ± 3.2 | +0.5 ± 3.6 | 0.952 | -1.1 ± 7.9 | +0.2 ± 3.0 | 0.635 |
| Sorbitol | -0.2 ± 1.1 | 0.2 ± 1.5 | 0.552 | +0.7 ± 0.9 | -0.1 ± 1.2 | 0.086 |
| Mannitol | -0.1 ± 1.2 | -0.3 ± 1.0 | 0.635 | +0.1 ± 0.3 | +0.1 ± 0.4 | 0.549 |
| Fructans | +0.03 ± 0.9 | 0.0 ± 0.9 | 0.941 | -0.5 ± 2.3 | +0.6 ± 2.2 | 0.220 |
| α-GOS | +0.2 ± 0.3 | -0.2 ± 0.5 | **0.032** | -0.8 ± 1.9 | -0.0 ± 0.8 | 0.608 |
| Oligosaccharides | +0.2 ± 1.0 | -0.2 ± 1.1 | 0.344 | -1.3 ± 4.1 | +0.6 ± 2.3 | 0.097 |
| Delta between pre-challenge (Visit 4 or Visit 6) and end of challenge (Visit 5 or Visit 7) values for responders (fiber-induced bloating) and non-responders (no induction of bloating) to fructan and α-GOS.  Modified intention-to-treat population used in analysis. Analyzed via unpaired t-test and presented as mean ± standard deviation (SD).  α-GOS, alpha-galactooligosaccharides; FODMAP, fermentable oligosaccharides, disaccharides, monosaccharides, and polyols | | | | | | |

| **Supplementary Table 3. Fermentations mechanisms (delta pre- to end of challenge) underpinning response to fiber** | | | | | | |
| --- | --- | --- | --- | --- | --- | --- |
| **Variable** | **Fructan responder delta (fiber-induced bloating)** | **Fructan non-responder delta**  **(no induction of bloating)** | **P** | α-**GOS responder delta (fiber-induced bloating)** | α-**GOS non-responder delta**  **(no induction of bloating)** | **P** |
| **Breath gas profiles** | | | | | | |
| Hydrogen | +1.0 [-1.0, 9.0] | +1.3 [-2.0, 14.6] | 0.993 | -12.5 [-36.4, 2.0] | +4.0 [-0.9, 8.3] | **0.008** |
| Methane | +0.1 [-0.1, 0.1] | +0.0 [-0.1, 0.1] | 0.638 | -20.0 [-32.1, 0.0] | 0.0 [-1.0, 0.0] | **0.008** |
| Oxygen | +22.5 [0.0, 30.0] | 0.0 [-3.7, 5.0] | 0.056 | 0.0 [0.0, 0.1] | 0.0 [-0.1, 0.0] | 0.484 |
| **SCFA concentrations** | | | | | | |
| Total SCFA | -11.4 [-27.2, 25.8] | -5.6 [-16.9, 11.8] | 0.965 | +44.1 [-16.8, -33.4] | +15.4 [-3.4, -29.6] | 0.948 |
| Acetate | -10.1 [-18.1, 10.4] | -3.4 [-10.7, 12.5] | 0.533 | +30.3 [-5.4, -23.4] | +9.6 [-4.0, -19.0] | 0.832 |
| Propionate | -1.0 [-6.0, 8.1] | -2.0 [-5.1, 1.9] | 0.372 | -3.6 [-5.4, 2.8] | -1.1 [-6.4, 6.5] | 0.591 |
| Butyrate | -0.1 [-1.7, 6.1] | -0.4 [-4.4, 2.8] | 0.422 | +11.4 [-3.1, -6.2] | +3.0 [0.0, -6.5] | 0.832 |
| Valerate | +0.6 [-0.6, 0.7] | -0.5 [-1.1, -0.1] | 0.256 | +0.2 [-0.6, -1.1] | +0.1 [-0.2, -1.1] | 0.544 |
| **BCFA concentrations** | | | | | | |
| Total BCFA | +1.4 [-0.7, 2.7] | -0.6 [-1.4, 0.4] | 0.053 | +0.5 [-0.2, -1.0] | -0.1 [-0.8, -1.7] | 0.571 |
| Iso-butyrate | -1.4 [-4.2, -0.5] | -0.2 [-0.6, 0.1] | **0.019** | +0.2 [-0.2, -0.4] | +0.0 [-0.2, -0.6] | 0.949 |
| Iso-valerate | +0.8 [-0.3, 1.6] | -0.4 [-0.9, 0.3] | 0.059 | +0.3 [0.0, -0.7] | -0.1 [-0.5, -1.1] | 0.478 |
| **SCFA:BCFA** | | | | | | |
| SCFA:BCFA | -8.6 [-14.3, 0.6] | +3.3 [-4.7, 13.6] | 0.074 | +3.1 [-0.8, -2.3] | +0.1 [-1.9, -5.5] | 0.429 |
| Delta between pre-challenge (Visit 4 or Visit 6) and end of challenge (Visit 5 or Visit 7) values for responders (fiber-induced bloating) and non-responders (no induction of bloating) to fructan and α-GOS.  mITT population used in analysis. Analyzed via Mann Whitney tests and presented as median [interquartile range (IQR)].  α-GOS, alpha-galactooligosaccharides; BCFA, branched-chain fatty acids; SCFA, short-chain fatty acids; SCFA:BCFA, ratio of short-chain to branched-chain fatty acids | | | | | | |

| **Supplementary Table 4. Clinical predictors of visceral hypersensitivity** | | | |
| --- | --- | --- | --- |
| **Variable** | **No visceral hypersensitivity** | **Visceral hypersensitivity** | **P*** |
| **IGQ** | | | |
| Bloating | 16.7 [5.8, 49.2] | 21.7 [15.0, 31.7] | 0.828 |
| Flatulence | 18.3 [10.0, 33.3] | 6.7 [6.7, 18.3] | **0.045** |
| Belching | 5.0 [0.0, 15.0] | 10.0 [0.0, 20.0] | 0.404 |
| Bad breath | 5.0 [0.0, 15.0] | 10.0 [0.0, 20.0] | 0.404 |
| Stomach rumbling | 10 [0.0, 27.5] | 5.0 [0.0, 22.5] | 0.594 |
| Difficult gas evacuation | 12.5 [0.0, 25.0] | 0.0 [0.0, 12.5] | 0.232 |
| Global score | 14.2 [4.6, 29.0] | 9.9 [6.1, 18.2] | 0.281 |
| Global score (except bloating) | 14.7 [3.7, 26.7] | 8.7 [4.8, 16.5] | 0.308 |
| **GSRS** | | | |
| Pain | 0.0 [0.0, 0.0] | 0.0 [0.0, 0.0] | 0.738 |
| Discomfort | 0.0 [0.0, 0.3] | 0.0 [0.0, 0.7] | 0.799 |
| Heartburn | 0.0 [0.0, 0.0] | 0.0 [0.0, 0.0] | >0.999 |
| Reflux | 0.0 [0.0, 0.0] | 0.0 [0.0, 0.0] | >0.999 |
| Nausea | 0.0 [0.0, 0.0] | 0.0 [0.0, 0.0] | >0.999 |
| Gurgling | 0.0 [0.0, 1.0] | 0.0 [0.0, 0.3] | 0.402 |
| Bloating | 0.7 [0.3, 1.0] | 0.3 [0.0, 1.0] | 0.623 |
| Belching | 0.0 [0.0, 1.0] | 0.0 [0.0, 0.0] | 0.149 |
| Flatulence | 0.7 [0.3, 1.0] | 0.3 [0.0, 0.7] | 0.112 |
| Constipation | 0.0 [0.0, 0.0] | 0.0 [0.0, 0.3] | 0.538 |
| Diarrhea | 0.0 [0.0, 0.0] | 0.0 [0.0, 0.0] | >0.999 |
| Loose stools | 0.0 [0.0, 0.3] | 0.0 [0.0, 0.0] | >0.999 |
| Hard stools | 0.0 [0.0, 0.0] | 0.0 [0.0, 0.3] | 0.793 |
| Urgency | 0.0 [0.0, 0.0] | 0.0 [0.0, 0.3] | 0.587 |
| Incomplete evacuation | 0.0 [0.0, 0.3] | 0.0 [0.0, 0.0] | >0.999 |
| Overall symptoms | 0.7 [0.0, 1.0] | 0.7 [0.0, 1.0] | 0.758 |
| **GSQ** | | | |
| % days with adequate relief | 100.0 [70.0, 100.0] | 100.0 [70.0, 100.0] | 0.752 |
| % days with inadequate relief | 0.0 [0.0, 30.0] | 0.0 [0.0, 30.0] | 0.752 |
| **BSFS** | | | |
| Form | 4.0 [3.5, 4.5] | 3.8 [2.9, 4.3] | 0.549 |
| Frequency | 1.3 [1.0, 2.0] | 1.7 [1.0, 2.3] | 0.236 |
| % firm | 0.0 [0.0, 0.0] | 0.0 [0.0, 40.0] | 0.635 |
| % normal | 100.0 [80.0, 100.0] | 100.0 [60.0, 100.0] | 0.574 |
| % loose | 0.0 [0.0, 0.0] | 0.0 [0.0, 0.0] | >0.999 |
| **Waist circumference** | | | |
| WC, *cm* | 78.0 [69.7, 89.0] | 72.7 [70.7, 77.7] | 0.508 |
| **Appetite** | | | |
| Hungry | -13.0 [-17.0, 2.0] | 4.0 [-4.0, 6.0] | **0.038** |
| Full | 1.0 [-20.0, 22.0] | -9.0 [-32.0, 5.0] | 0.426 |
| Satisfied | 0.0 [-7.0, 16.0] | -9.0 [-11.0, 0.0] | 0.583 |
| Eat now | -2.0 [-26.0, 5.0] | -2.0 [-7.0, 7.0] | 0.337 |
| Baseline (Visit 3) values between participants without visceral hypersensitivity (non-VH) and visceral hypersensitivity (VH).  Modified per-protocol population population used in analysis. *Analyzed via unpaired t-test and presented as mean ± standard deviation (SD) for normal distributed data and analyzed via Mann Whitney tests and presented as median [interquartile range (IQR)] if data assessed to be non-normally distributed.  BSFS, Bristol Stool Form Scale; GSQ, Gastrointestinal Symptom Question; GSRS, Gastrointestinal Symptom Rating Scale; WC, waist circumference. | | | |

| **Supplementary Table 5. Nutritional predictors of visceral hypersensitivity** | | | |
| --- | --- | --- | --- |
| **Variable** | **No visceral hypersensitivity** | **Visceral hypersensitivity** | **P** |
| **Nutritional intake (per day)** | | | |
| Energy, *kcal* | 1549.0 [1104.0, 1822.0] | 1617.0 [1258.0, 1825.0] | >0.999 |
| Protein, *g* | 67.8 [64.3, 119.6] | 64.7 [60.1, 75.0] | 0.349 |
| Fat, *g* | 71.7 [44.5, 82.0] | 62.5 [53.5, 82.1] | 0.918 |
| Saturated fat, *g* | 20.2 [ 13.8, 26.5] | 18.9 [15.4, 25.7] | 0.918 |
| Carbohydrates, *g* | 147.6 [111.3, 191.9] | 147.9 [124.4, 187.7] | 0.725 |
| Starch, *g* | 94.8 [52.2, 118.8] | 92.5 [71.5, 108.9] | 0.835 |
| Sugars, *g* | 66.1 [43.5, 83.8] | 55.4 [ 38.7, 72.1] | 0.426 |
| Fiber, *g* | 19.6 [17.6, 27.4] | 22.1 [12.8, 35.0] | 0.557 |
| Non-starch polysaccharide, *g* | 15.0 [10.5, 19.4] | 16.1 [8.5, 26.2] | 0.654 |
| Calcium, *g* | 669.0 [487.9, 964.0] | 536.9 [485.4, 756.5] | 0.688 |
| Iron, *mg* | 10.4 [8.4, 18.8] | 10.9 [7.5, 15.8] | 0.315 |
| Zinc, *mg* | 9.9 [7.5, 21.0] | 7.3 [6.8, 9.5] | **0.037** |
| Sodium, *mg* | 1726.0 [999.6, 2116.0] | 2313.0 [1596.0, 2608.0] | 0.317 |
| Potassium, *mg* | 2442.0 [1910.0, 3588.0] | 2481.0 [1678.0, 3440.0] | 0.756 |
| Phosphorus, *ug* | 1078.0 [827.5, 1192.0] | 985.4 [798.1, 1274.0] | 0.809 |
| Magnesium, *mg* | 296.9 [193.9, 381.1] | 356.0 [187.4, 462.8] | 0.958 |
| Iodine, *ug* | 195.5 [98.4, 260.1] | 71.5 [57.8, 112.6] | 0.115 |
| Selenium, *ug* | 60.7 [41.6, 114.5] | 52.6 [35.0, 59.4] | 0.099 |
| Vitamin A, *ug* | 568.7 [223.3, 1043.0] | 761.1 [238.0, 2309.0] | 0.605 |
| Vitamin C, *ug* | 111.1 [65.9, 188.2] | 86.0 [58.5, 213.2] | 0.654 |
| Vitamin D, *ug* | 4.9 [2.5, 8.0] | 4.2 [2.4, 6.5] | 0.654 |
| Vitamin B9, *ug* | 260.8 [184.4, 495.8] | 270.6 [211.7, 372.2] | 0.654 |
| Vitamin B12, *ug* | 5.3 [3.6, 11.5] | 4.34 [2.6, 8.7] | 0.251 |
| **FODMAP intake** | | | |
| Total FODMAPs, *g* | 17.9 [6.5, 20.8] | 9.0 [5.7, 15.3] | 0.061 |
| Excess fructose, *g* | 1.0 [0.7, 3.4] | 1.0 [0.5, 1.2] | 0.524 |
| Lactose, *g* | 7.3 [0.7, 14.6] | 3.7 [1.0, 6.9] | 0.146 |
| Sorbitol, *g* | 0.8 [0.2, 2.6] | 0.5 [0.0, 1.2] | 0.151 |
| Mannitol, *g* | 0.2 [0.1, 1.0] | 0.1 [0.1, 0.5] | 0.639 |
| Fructans, *g* | 2.0 [1.6, 3.2] | 2.0 [1.5, 3.7] | 0.704 |
| α-GOS, *g* | 0.6 [0.3, 1.0] | 0.6 [0.1, 1.2] | 0.932 |
| Oligosaccharides, *g* | 2.8 [2.0, 3.8] | 3.4 [1.5, 5.3] | 0.561 |
| Baseline (Visit 3) values between participants without visceral hypersensitivity (non-VH) and with visceral hypersensitivity (VH)  Modified per-protocol population population used in analysis. Analyzed via Mann Whitney tests and presented as median [interquartile range (IQR)].  FODMAP, fermentable oligosaccharides, disaccharides, monosaccharides, and polyols; α-GOS, alpha-galactooligosaccharides | | | |

| **Supplementary Table 6. Fermentation predictors of visceral hypersensitivity** | | | |
| --- | --- | --- | --- |
| **Variable** | **Non-VH** | **VH** | **P** |
| **Breath gas profiles** | | | |
| Hydrogen | 9.3 [5.0, 18.0] | 5.7 [2.0, 14.3] | 0.657 |
| Methane | 0.0 [0.0, 26.0] | 0.0 [0.0, 45.0] | 0.990 |
| Oxygen | 14.6 [14.5, 14.7] | 14.5 [14.4, 14.6] | 0.421 |
| **SCFA concentrations** | | | |
| Total SCFA*, umol/g* | 44.9 [24.4, 90.2] | 70.3 [38.6, 102.4] | 0.318 |
| Acetate*, umol/g* | 25.9 [15.1, 58.2] | 38.9 [24.6, 75.8] | 0.330 |
| Propionate*, umol/g* | 8.7 [4.4, 13.1] | 12.5 [8.0, 20.2] | 0.211 |
| Butyrate*, umol/g* | 8.7 [3.8, 16.7] | 11.5 [4.1, 13.6] | 0.549 |
| Valerate*, umol/g* | 0.9 [0.5, 1.5] | 1.4 [1.0, 2.8] | 0.064 |
| **BCFA concentrations** | | | |
| Total BCFA | 2.6 [1.4, 3.9] | 2.2 [1.8, 3.0] | 0.940 |
| Iso-butyrate | 0.96 [0.6, 1.53] | 1.1 [0.7, 1.2] | 0.710 |
| Iso-valerate | 1.7 [0.8, 2.4] | 1.3 [1.0, 1.8] | 0.868 |
| **SCFA:BCFA** | | | |
| SCFA:BCFA | 10.9 [9.3, 44.2] | 27.9 [14.8, 36.3] | 0.412 |
| Baseline (V3) values between participants without visceral hypersensitivity (non-VH) and with visceral hypersensitivity (VH)  Modified per-protocol population population used in analysis. Analyzed via Mann Whitney tests and presented as median [interquartile range (IQR)].  BCFA, branched-chain fatty acids; SCFA, short-chain fatty acids; SCFA:BCFA, ratio of short-chain to branched-chain fatty acids. | | | |

| **Supplementary Table 7. Significant CAZys associated with visceral hypersensitivity** | | | | | | | | |
| --- | --- | --- | --- | --- | --- | --- | --- | --- |
| **CAZy abbreviation** | **CAZy name** | **Module name** | **Mean normalized signal** | **Log2 fold change** | **Standard error of log2 fold change** | **Test statistic** | **P-value** | **Adjusted p-value** |
| **GH89** | Glycoside Hydrolase Family 89 | Glycoside Hydrolase | 161.6 | 2.5 | 0.6 | 4.3 | <0.001 | 0.003 |
| **CBM26** | Carbohydrate-Binding Module Family 26 | Carbohydrate-Binding Module | 1820.6 | 3.3 | 0.9 | 3.8 | <0.001 | 0.010 |
| **GH13_42** | Glycoside Hydrolase Family 13 / Subf 42 | Glycoside Hydrolase | 200.3 | 4.2 | 1.2 | 3.6 | <0.001 | 0.010 |
| **GT20** | GlycosylTransferase Family 20 | GlycosylTransferase | 62.8 | 3.2 | 0.9 | 3.7 | <0.001 | 0.010 |
| **GH13_19** | Glycoside Hydrolase Family 13 / Subf 19 | Glycoside Hydrolase | 82.3 | 2.9 | 0.9 | 3.4 | <0.001 | 0.017 |
| **GH29** | Glycoside Hydrolase Family 29 | Glycoside Hydrolase | 530.3 | 1.2 | 0.3 | 3.4 | <0.001 | 0.017 |
| **GH26** | Glycoside Hydrolase Family 26 | Glycoside Hydrolase | 216.6 | 1.6 | 0.5 | 3.3 | <0.001 | 0.019 |
| **GH13_28** | Glycoside Hydrolase Family 13 / Subf 28 | Glycoside Hydrolase | 206.8 | 2.8 | 0.9 | 3.1 | 0.002 | 0.035 |
| **GH99** | Glycoside Hydrolase Family 99 | Glycoside Hydrolase | 31.8 | 2.3 | 0.7 | 3.1 | 0.002 | 0.035 |
| **GH27** | Glycoside Hydrolase Family 27 | Glycoside Hydrolase | 525.3 | 1.9 | 0.6 | 3.0 | 0.002 | 0.035 |
| **GH43_5** | Glycoside Hydrolase Family 43 / Subf 5 | Glycoside Hydrolase | 84.0 | 4.9 | 1.7 | 2.9 | 0.003 | 0.044 |
| **PL11** | Polysaccharide Lyase Family 11 | Polysaccharide Lyase | 157.0 | 2.2 | 0.7 | 2.9 | 0.003 | 0.044 |
| Difference in expression of CAZys between those with visceral hypersensitivity and without visceral hypersensitivity at baseline (Visit 3)  Modified per-protocol population; population used in analysis. Analyzed using DESeq2. A positive log2FoldChange corresponds to higher expression in the visceral hypersensitivity group.  CAZy, Carbohydrate-Active enZyme; CBM, Carbohydrate-Binding Module; GH, Glycoside Hydrolase; GT, GlycosylTransferase; PL, Polysaccharide Lyase. | | | | | | | | |

**Gut Microbiome Composition and Function, Diet and Clinical Factors in Relation to Fermentable Carbohydrate-Induced Bloating: A Double-Blind, Randomized, Crossover Trial**

**Supplementary Figures**

**Supplementary Figure 1. Heatmap of significant mOTUs and genera for fructan responders (those experiencing fiber-induced bloating)**

DESeq2 results; *too many zero values (only outlier and zero points in boxplots); ^#^very low signal (DESeq2 baseMean<10 or filtered out for 2 of the 3 performed analyses).

**Supplementary Figure 2. Heatmap of significant GMMs for fructan response responders (those experiencing fiber-induced bloating)**

DESeq2 results; *too many zero values; ^#^very low signal (DESeq2 baseMean<10 or filtered out for 2 of the 3 performed analyses).

**Supplementary Figure 3. Heatmap of significant CAZymes for fructan response responders (those experiencing fiber-induced bloating)**

**Supplementary Figure 4. Heatmap of significant mOTUs and genera for GOS response responders (those experiencing fiber-induced bloating)**

DESeq2 results; *too many zero values; ^#^very low signal (DESeq2 baseMean<10 or filtered out for 2 of the 3 performed analyses).

**Supplementary Figure 5. Boxplot of triacylglycerol degradation by GOS responder status, comparing GOS responders (fiber-induced bloating) and GOS non-responders (no induction of bloating)**

*******

***padj<0.001 with DESeq2 analysis.

**Supplementary Figure 6. Boxplots of fructans related to GOS degradation by GOS responder status, comparing GOS responders (fiber-induced bloating) and GOS non-responders (no induction of bloating)**

p≥0.05 for all comparisons with DESeq2 analysis.

**Supplementary Figure 7. Boxplot of pre-challenge *Faecalibacterium/Bacteroides* ratio by responder status, comparing responders (fiber-induced bloating) and non-responders (no induction of bloating)**

**^$$^** p<0.01, **^$^**p<0.05 with Mann-Whitney tests for pre-challenge values

**Supplementary Figure 8. Boxplots of pre-challenge p-cresol stool concentrations by fructan responder status, comparing fructan responders (fiber-induced bloating) and fructan non-responders (no induction of bloating)**

**P = 0.009**

**Fructan
Non-responder**

**Fructan Responder**

**References**

1.	Drossman, D.A. and W.L. Hasler, *Rome IV—Functional GI Disorders: Disorders of Gut-Brain Interaction.* Gastroenterology, 2016. **150**(6): p. 1257-1261.

2.	Wilson, B., et al., *Prebiotics in irritable bowel syndrome and other functional bowel disorders in adults: a systematic review and meta-analysis of randomized controlled trials.* The American Journal of Clinical Nutrition, 2019. **109**(4): p. 1098-1111.

3.	Suarez, F.L., et al., *Gas production in human ingesting a soybean flour derived from beans naturally low in oligosaccharides.* Am J Clin Nutr, 1999. **69**(1): p. 135-9.

4.	Skodje, G.I., et al., *Fructan, Rather Than Gluten, Induces Symptoms in Patients With Self-Reported Non-Celiac Gluten Sensitivity.* Gastroenterology, 2018. **154**(3): p. 529-539.e2.

5.	Mego, M., et al., *Metabolic adaptation of colonic microbiota to galactooligosaccharides: a proof-of-concept-study.* 2017. **45**(5): p. 670-680.

6.	Mego, M., et al., *Colonic gas homeostasis: Mechanisms of adaptation following HOST-G904 galactooligosaccharide use in humans.* 2017. **29**(9): p. e13080.

7.	Erickson, J., et al., *Gastrointestinal tolerance of low FODMAP oral nutrition supplements in healthy human subjects: a randomized controlled trial.* Nutrition Journal, 2017. **16**(1): p. 35.

8.	Hustoft, T.N., et al., *Effects of varying dietary content of fermentable short-chain carbohydrates on symptoms, fecal microenvironment, and cytokine profiles in patients with irritable bowel syndrome.* 2017. **29**(4): p. e12969.

9.	Depeint, F., et al., *Prebiotic evaluation of a novel galactooligosaccharide mixture produced by the enzymatic activity of Bifidobacterium bifidum NCIMB 41171, in healthy humans: a randomized, double-blind, crossover, placebo-controlled intervention study2.* The American Journal of Clinical Nutrition, 2008. **87**(3): p. 785-791.

10.	Bonnema, A.L., et al., *Gastrointestinal Tolerance of Chicory Inulin Products.* Journal of the American Dietetic Association, 2010. **110**(6): p. 865-868.

11.	Josefsson, A., H. Törnblom, and M. Simrén, *Type of Rectal Barostat Protocol Affects Classification of Hypersensitivity and Prediction of Symptom Severity in Irritable Bowel Syndrome.* Journal of Neurogastroenterology and Motility, 2022. **28**(4): p. 630-641.

12.	Chen, S., et al., *fastp: an ultra-fast all-in-one FASTQ preprocessor.* Bioinformatics, 2018. **34**(17): p. i884-i890.

13.	Milanese, A., et al., *Microbial abundance, activity and population genomic profiling with mOTUs2.* Nature Communications, 2019. **10**(1): p. 1014.

14.	Li, J., et al., *An integrated catalog of reference genes in the human gut microbiome.* Nature Biotechnology, 2014. **32**(8): p. 834-841.

15.	Reade, S., et al., *Optimisation of Sample Preparation for Direct SPME-GC-MS Analysis of Murine and Human Faecal Volatile Organic Compounds for Metabolomic Studies.* J Anal Bioanal Tech, 2014. **5**.

16.	Cox, S.R., et al., *Fermentable Carbohydrates [FODMAPs] Exacerbate Functional Gastrointestinal Symptoms in Patients With Inflammatory Bowel Disease: A Randomised, Double-blind, Placebo-controlled, Cross-over, Re-challenge Trial.* J Crohns Colitis, 2017. **11**(12): p. 1420-1429.

17.	Shepherd, S.J., et al., *Dietary triggers of abdominal symptoms in patients with irritable bowel syndrome: randomized placebo-controlled evidence.* Clin Gastroenterol Hepatol, 2008. **6**(7): p. 765-71.

18.	Enck, P. and S. Klosterhalfen, *Placebo Responses and Placebo Effects in Functional Gastrointestinal Disorders.* Front Psychiatry, 2020. **11**: p. 797.

19.	Love, M.I., W. Huber, and S. Anders, *Moderated estimation of fold change and dispersion for RNA-seq data with DESeq2.* Genome Biol, 2014. **15**(12): p. 550.
